# Sleep and participation in extracurricular activities moderate associations between low SES and brain structure and functional connectivity

**DOI:** 10.1016/j.dcn.2026.101827

**Published:** 2026-09-28

**Authors:** Juliette Tinch, Phoebe Thomson, Divyangana Rakesh

**Affiliations:** Department of Neuroimaging, Institute of Psychology, Psychiatry and Neuroscience, King's College London, London, UK

**Keywords:** Socioeconomic status, Adolescence, Brain structure, Resting state functional connectivity, Income, Neighborhood disadvantage, Moderation, Sleep duration, Extracurriculars

## Abstract

Household and neighborhood socioeconomic status (SES) have consistently been associated with both brain structure and functional connectivity in adolescence. However, there is considerable heterogeneity in brain outcomes among youth from disadvantaged backgrounds, which suggests that other factors—including aspects of one’s lifestyle—likely play a role by moderating these associations. In a large sample of ~10,000 early adolescents from the baseline assessment of the Adolescent Brain Cognitive Development study (aged 9–10 years), we tested whether sleep and participation in physical and non-physical extracurricular activities moderate associations of household and neighborhood disadvantage with brain structure and functional connectivity. Higher sleep duration was linked to weaker associations between both household and neighborhood disadvantage and a range of structural and functional brain measures, whereas extracurricular engagement had more selective moderating effects on the structure of sensory regions. Our findings identify sleep duration and extracurricular engagement as markers of variability in SES-brain associations, underscoring the need for longitudinal and intervention studies to determine whether these factors can mitigate disparities in brain outcomes among youth from disadvantaged backgrounds.

## Highlights

• Low income and neighborhood disadvantage are associated with adolescent brain structure and functional connectivity.
• Higher sleep duration and participation in extracurricular activities moderate these associations.
• Sleep duration has widespread moderating effects while extracurriculars show more selective effects for sensory systems.
• Variability in brain outcomes in disadvantaged contexts may arise due to differences in sleep and extracurricular participation
• Future studies should test whether interventions targeting sleep and extracurricular participation promote resilience.

## 1 Introduction

Socioeconomic status (SES) is a multidimensional construct which encompasses access to financial and non-financial resources at both the household and neighborhood level (Duncan and Magnuson, 2012, Farah, 2017). Higher SES is associated with more favorable life outcomes, including better physical and mental health, higher cognitive performance, and higher academic achievement (Farah, 2017, Hackman et al., 2015, Noble et al., 2007). SES is also linked to variation in brain structure and function (Rakesh and Whittle, 2021), which may underlie the SES-cognition relationship (Farah, 2018, Noble et al., 2015, Rakesh et al., 2021, Rosen et al., 2025). These associations have consistently been reported during adolescence, a period marked by dynamic structural and functional brain development (Ernst et al., 2015, Norbom et al., 2021, Sanders et al., 2023, Stevens, 2016, Tamnes et al., 2017). Importantly, despite consistent group-level effects, there is considerable individual heterogeneity in neural outcomes among youth from low SES backgrounds. Indeed, it has recently been shown that children from structurally disadvantaged and marginalized backgrounds show markedly greater within-group variability in neurodevelopment than their peers from more advantaged backgrounds (Bottenhorn et al., 2024). It is therefore possible that other—potentially modifiable—factors contribute to this variability by moderating the effects of low SES on the brain. Identifying such factors could inform future research aimed at promoting healthy development in low socioeconomic contexts.

SES—including family income and neighborhood disadvantage—is associated with brain structure and connectivity in youth (Rakesh and Whittle, 2021). In particular, higher household and neighborhood SES are consistently associated with higher total grey matter volume and surface area (Brito et al., 2018, Carrick et al., 2026, Hackman et al., 2021, Hanson et al., 2013, Judd et al., 2020, Luby et al., 2013, Mackey et al., 2015, McDermott et al., 2019, Merz et al., 2022, Noble et al., 2015, Norbom et al., 2024, Rakesh et al., 2022); however, evidence for associations with average cortical thickness is less consistent, with reports of both positive associations (McDermott et al., 2019; Miller et al., 2022; Tsomokos et al., 2025) and null findings (e.g., Brito et al., 2018). Beyond global measures, SES-related differences have been observed throughout the cortex, with the most consistent effects found in frontal regions (Rakesh and Whittle, 2021).

Family income and neighborhood disadvantage are also associated with resting state functional connectivity (rsFC), with higher income-to-needs and lower neighborhood poverty generally linked to greater within-network connectivity and network segregation (Michael et al., 2023, Rakesh et al., 2021). However, the direction of effects seems to vary by network. For example, higher household and neighborhood SES are associated with *lower* within-network rsFC and segregation in sensorimotor networks (Michael et al., 2024, Rakesh et al., 2021). A similar difference in directionality has also been observed for between-network connectivity. For example, higher neighborhood disadvantage is associated with lower connectivity between sensorimotor and higher-order networks but with higher connectivity *within* sensorimotor networks (Rakesh, Seguin, et al., 2021). Together, these findings provide consistent evidence of associations of family income and neighborhood SES with brain structure and function during adolescence. Importantly, the direction and magnitude of these associations varies across brain systems, and there is substantial variability in brain outcomes among youth from disadvantaged backgrounds (Bottenhorn et al., 2024). This lack of uniform vulnerability highlights the importance of identifying markers of such variability while taking system-level differences into account.

Indeed, although income and neighborhood disadvantage are associated with brain outcomes, positive environmental and psychological factors may moderate these effects. For example, household and structural factors—such as positive parenting and school environments—as well as temperamental effortful control, have been shown to buffer associations of income and neighborhood SES with brain structure and connectivity (Brody et al., 2019, Rakesh et al., 2021, Rakesh et al., 2021, Whittle et al., 2017). Although investigated in fewer studies, modifiable lifestyle factors are also potential candidates, particularly longer sleep duration and participation in physical and non-physical extracurricular activities which are known to promote higher cognitive ability, academic performance, and better mental health (Astill et al., 2012, Biddle et al., 2019, Boelens et al., 2022, de Greeff et al., 2018, Hosker et al., 2019, Ishiguro et al., 2023, Musshafen et al., 2021, Pascoe et al., 2020, Román-Caballero et al., 2022, Zarazaga-Peláez et al., 2024, Zheng et al., 2025). Importantly, these benefits have been observed in the context of disadvantage (El-Sheikh et al., 2022, Heath et al., 2022, Kleszczewska et al., 2018, Morris, 2015, Pan et al., 2022, Rakesh et al., 2024). No studies, however, have examined the role of such factors in the association of household and neighborhood disadvantage with children’s brain structure and function. Given that sleep and extracurricular participation are modifiable factors that are directly intervenable (Albakri et al., 2021, Jago et al., 2015), research on whether they moderate the SES-brain association has the capacity to inform longitudinal and intervention studies aimed at reducing socioeconomic disparities in neurodevelopment.

Finally, as different indicators of SES capture distinct aspects of a child’s environment (Duncan and Magnuson, 2012), it is possible that moderating effects do not follow a one-size-fits-all pattern. At the household level, low income may reflect material deprivation, influencing aspects such as housing, healthcare, childcare, cognitive stimulation and nutrition (Duncan and Magnuson, 2012, Duncan and Magnuson, 2003). Low neighborhood SES, on the other hand, may capture distinct social and physical chronic stressors such as exposure to crime, violence and pollutants as well as resource-based factors such as access to libraries and school quality (Evans, 2004, Hyde et al., 2022). Such factors may be particularly relevant during the transition from childhood to adolescence when children begin engaging independently with their neighborhoods (Boardman and Saint Onge, 2005, Leventhal and Brooks-Gunn, 2000, Sampson et al., 2002). Critically, these distinct environmental contexts have overlapping yet distinct associations with both neural and non-neural outcomes (Carroll et al., 2024, Noble and Giebler, 2020, Rakesh et al., 2021). As such, moderating effects may be specific to one or the other SES indicator. For example, adolescents from low-income households, where sleep may be disrupted due to stress (Fronberg et al., 2022, Philbrook et al., 2020) and cognitive stimulation may be reduced (Rakesh et al., 2024, Rosen et al., 2020), may benefit more from adequate sleep duration and cognitively enriching non-physical extracurricular activities. In contrast, adolescents from disadvantaged neighborhoods who have limited access to safe outdoor spaces (Crawford et al., 2008, Franzini et al., 2010, Marquet et al., 2019, McKenzie et al., 2013) may benefit more from physical extracurricular activities. This is particularly relevant given that these SES indicators are only moderately correlated with one another, and individuals may experience some forms of socioeconomic disadvantage but not others (Farah, 2017). Consequently, examining both household and neighborhood disadvantage is needed to achieve a more nuanced and accurate understanding of moderating factors.

To address these crucial gaps, the present study aimed to investigate whether sleep duration and participation in physical or non-physical extracurricular activities moderate the relationship between household and neighborhood disadvantage—operationalized as income-to-needs ratio and area-level deprivation—and global brain structure and function in early adolescence. Drawing on prior work showing differential associations between SES and sensorimotor versus higher-order networks (Michael et al., 2024, Rakesh et al., 2021), we also examined these systems separately in secondary analyses. We hypothesized that greater sleep duration and higher levels of engagement in extracurricular activities—both physical and non-physical—would be associated with weaker relationships between SES and brain outcome measures. We further expected that the strength of these moderating effects may vary between SES indicators. Specifically, sleep duration and participation in non-physical extracurricular activities may show stronger moderating effects in the context of lower income, whereas physical activity may be relevant to the context of high neighborhood disadvantage. Although prior work indicated variation in the direction of the association between SES and different brain systems, we did not formulate specific hypotheses regarding differential effects of lifestyle factors given the dearth of research on this topic.

## 2 Methods

### 2.1 Pre-registration

This study was preregistered on the Open Science Framework (osf.io/3ju9e). Any deviations from the original plan are fully described.

### 2.2 Participants

We used baseline data from release 6.0 of the ongoing Adolescent Brain Cognitive Development (ABCD) study (https://abcdstudy.org/), a large-scale study assessing the psychological and neurobiological development of over 11,500 children aged 9–11 at baseline. Participants were principally recruited through public and private elementary schools in 21 geographically and demographically diverse areas across the US. The ABCD sampling strategy maximized the cohort’s representation of U.S. demographic and socioeconomic groups by oversampling schools with higher percentages of specific underrepresented groups (e.g., African Americans and adolescents living in rural areas) (Garavan et al., 2018). Ethical approval for all ABCD procedures has been granted by the central Institutional Review Board of the University of California. Any participants missing SES or modifiable lifestyle factors data as well as those with scans not meeting brain imaging quality standards were excluded from analyses. See Table 1 for information on the analytic sample.

**Table 1:** Demographic and descriptive statistics. Demographic information has been provided for the largest possible sample size (those with area deprivation index [ADI] and usable structural data). Available data and sample sizes for specific analyses were as follows: (1) ADI and usable structural data: N = 10,470 (0.06% missing extracurricular activity, 0.04% missing sleep); (2) income-to-needs ratio (INR) and usable structural data: N = 10,171 (0.02% missing extracurricular activity, 0.00% missing sleep); (3) ADI and usable functional connectivity data: N = 8731 (0.06% missing extracurricular activity, 0.05% missing sleep); (4) INR and usable functional connectivity data: N = 8499 (0.02% missing extracurricular activity, 0.00% missing sleep). SD = standard deviation. Differences in demographic characteristics between participants included in the analyses and those excluded due to missing sMRI or rs-fMRI data are provided in the Supplementary Materials. Overall, participants missing MRI data were more likely to be male, younger, from lower-SES backgrounds, and, in the rs-fMRI sample, to report shorter sleep duration (Tables S4 and S5).

Characteristic	N or Mean (SD)	[Min, Max]
No. of participants (Female %)	10,470 (48%)	
Age (years)	10.0 (0.6)	[8.3, 11.3]
Income-to-reeds ratio	3.7 (2.4)	[0.1, 12.4]
ADI	39.8 (26.8)	[1, 100]
Average parental education (years)	15.1 (2.6)	[3, 22]
Physical extracurricular activity (hours per week)	6.7 (6.8)	[0, 76.8]
Non-physical extracurricular activity (hours per week)	1.8 (3.2)	[0, 36]
Sleep duration (hours)	9.0 (1.1)	[5, 10]

### 2.3 Measures of SES

#### 2.3.1 Neighborhood disadvantage

Neighborhood Disadvantage was operationalized using the Area Deprivation Index (ADI), a composite measure of neighborhood socioeconomic disadvantage with 17 metrics including median income, education, employment, and home value and quality. Participants’ ADI was determined using the participant’s primary address, with neighborhoods ranked as a national percentile. Higher ADI values represent higher neighborhood disadvantage.

#### 2.3.2 Household SES

Household SES was operationalized using the income-to-needs ratio, which was calculated by dividing the median value of the total household income band by the 2017 federal poverty guideline for the respective household size (U.S. Department of Health & Human Services, 2017). A ratio of one represents being at the poverty threshold, and values greater than and less than one indicate being above and below the threshold, respectively. Household income bands have been provided in the Supplement. At the household level, SES was indexed using income-to-needs ratio because one of the primary aims of the study was to examine whether modifiable lifestyle factors moderate associations between material disadvantage at the household level and adolescent brain outcomes. Income-to-needs ratio directly indexes proximal material conditions and resource constraints that are theoretically relevant to the lifestyle factors examined here, including access to extracurricular opportunities and sleep-related environmental stressors (Fronberg et al., 2022, Philbrook et al., 2020, Snellman et al., 2015). In contrast, parental educational attainment may influence neurodevelopment through partially distinct pathways, such as the home cognitive environment and parenting practices. Retaining these indicators separately therefore allowed us to more specifically examine moderation of associations with household material disadvantage, rather than a broader composite SES construct reflecting multiple socioeconomic dimensions. Consistent with our preregistered plan, parental educational attainment was included as a covariate given its association with income-to-needs ratio, neighborhood disadvantage, and brain outcomes. In addition, income was also prioritized given its direct relevance to social and economic policy interventions that target material resources during childhood and adolescence (Duncan et al., 2011). Although not preregistered, results for parental educational attainment controlling for income-to-needs have been provided in the supplement for completeness and to facilitate future meta-analytic work (Table S1). Finally, we conducted a non-preregistered analysis using a household SES composite measure derived from a principal component analysis of INR and parental educational attainment, as composite SES constructs may reduce measurement error (National Center for Education Statistics, 2012). Results for these models have been provided in the Supplement (Table S2).

### 2.4 Measures of brain structure and function

Participants’ neuroimaging took place at one of 21 sites across the United States with a harmonized protocol (3 T Siemens, Ge or Philips MRI scanners with a 32-channel head coil). 3D T1-weighted structural images were processed using cortical surface reconstruction in FreeSurfer version 7.1.1 and the Desikan-Killiany atlas for cortical parcellation (Desikan et al., 2006). In our main analysis, we used three global brain metrics—average cortical thickness, total brain volume, and total surface area. Regional brain measures show greater variability across scan types, particularly cortical thickness which is most inconsistent in frontal and temporal cortices (Knussmann et al., 2022). Global brain metrics reduce the impact of these regional variabilities, resulting in a higher signal-to-noise ratio (Hagler et al., 2019;Knussmann et al., 2022). Global metrics have also shown to have high test-retest reliability within individuals scanned repeatedly at the same site or after scanner relocation, and within individuals across scanners (Knussmann et al., 2022; Melzer et al., 2020). This is particularly relevant to the current study, as the ABCD dataset includes MRI data from multiple sites and scanner manufacturers (Hagler et al., 2019). Additionally, we further distinguished between 1) sensory (i.e., auditory and visual), 2) somatosensory/motor, and 3) higher-order association regions (the categorization of cortical regions is provided in the Supplement), as income may be associated with the structure of these regions in distinct ways. As such, we used a total of 12 structural MRI variables: average cortical thickness, total volume, and total surface area, each additionally computed for sensory, somatosensory/motor, and higher-order association cortices.

For functional connectivity variables, participants completed four 5-minute resting-state functional MRI (rs-fMRI) scans with eyes open to ensure a minimum of 8 min of usable resting-state data after concatenation and quality control. After preprocessing (Hagler et al., 2019), fMRI time courses were projected onto FreeSurfer’s cortical surface reconstruction, and within-network and between-network connectivity values for 12 predefined rsFC networks were computed using the Gordon parcellation scheme (Gordon et al., 2014). We used within-network and between-network connectivity calculated 1) on average across all 12 networks, and 2) separately for higher-order, somatosensory/motor, and sensory networks. Within- and between-network connectivity were analyzed separately as they follow different developmental trajectories; within-network connectivity mainly increases throughout development, whereas between-network connectivity is thought to decrease, reflecting a balance between segregation and integration (Ernst et al., 2015, Michael et al., 2023). This resulted in a total of 11 functional connectivity variables: 1) average between-network rsFC, 2) average within-network rsFC, 3) pair-wise connectivity between higher-order, somatosensory/motor, and sensory networks (n = 6 variables), and 4) average within-network rsFC for higher-order, somatosensory/motor, and sensory networks (n = 3 variables).

### 2.5 Measures of modifiable lifestyle factors

#### 2.5.1 Sleep duration

Sleep duration was assessed using an item from the parent-reported Sleep Disturbance Scale for Children, a scale in which parents report on their child’s sleep behavior over the past 6 months (Bruni et al., 1996, Ferreira et al., 2009). Caregivers were asked “How many hours of sleep does your child get on most nights?” and could choose one of five options: 9–11, 8–9, 7–8, 5–7, less than 5 h.

#### 2.5.2 Physical extracurricular activities and non-physical extracurricular activities

Extracurricular activity was assessed using the ABCD Study Sports and Activities Involvement Questionnaire (Huppertz et al., 2016), where parents report the activities their child participated in. Weekly duration of each activity was calculated by multiplying the reported number of sessions per week by the duration of each session. To capture potentially distinct effects of different activity types (Cabane et al., 2016), two variables were derived: total weekly duration of physical extracurricular activities and total weekly duration of non-physical extracurricular activities obtained by summing across all activities in each category. Physical activities included sports such as ballet, basketball, swimming, and yoga. Non-physical activities included musical instruments, arts such as drawing and theatre, competitive games and hobbies. A full list of the activities has been provided in the Supplement. Of note, inspection of the data revealed extreme values of duration of physical extracurricular activity that exceeded a plausible range, including a maximum of 76.8 h per week (≈11 h per day) which surpasses levels typically seen even in elite athletes (Saavedra et al., 2018). Although not pre-registered, to ensure that outliers did not bias our estimates, we winsorized the physical activity variable at the 98th percentile prior to modeling. Extracurricular non-physical activity was left unwinsorized given its lower maximum of 36 h per week. For transparency, we report results using unwinsorized physical activity in the Supplement (Tables S3).

### 2.6 Statistical Methods

We used linear mixed effect models (*lmer* package in R version 2024.12.1) to evaluate whether the modifiable factors sleep, physical activity, and non-physical extracurricular activities moderate the association between SES and adolescent brain structure and functional connectivity. To this end, we modeled brain variables as the outcome and income-to-needs or ADI as the predictor. To test the moderating role of sleep and extracurricular activity engagement, we modeled interaction terms between SES indicators (i.e., income-to-needs and ADI, in separate models) and each modifiable factor. Each combination of SES, protective factor, and brain outcome was estimated separately. Model equations have been provided in the Supplement.

Covariates included sex, age, mean framewise displacement (for rsFC variables), scanner model, and parental educational attainment. Parental educational attainment was included as a confounder to account for its association with family income, the home environment, neighborhood SES, as well as brain outcomes (Duncan and Magnuson, 2012, Duncan and Magnuson, 2003, Rakesh et al., 2022, Rakesh et al., 2021). Family ID was modelled as a random effect. In sensitivity analyses, we also included site as a random effect. Although not preregistered, we conducted additional sensitivity analyses. First, we used unwinsorized data for physical extracurricular activity engagement. Second, we fitted a quadratic term for sleep and its interaction with SES to investigate potential nonlinear moderation effects. SES indicators, modifiable factors, brain measures, age and parental educational attainment were scaled prior to analysis to obtain standardized estimates. Histograms, a correlation matrix of predictor variables, and joint distributions of lifestyle factors across household and neighborhood disadvantage can be found in the Supplement. To correct for multiple comparisons and minimize the rate of false positives, we applied a false discovery rate (FDR) correction (pFDR <.05) (Benjamini and Hochberg, 1995) across models for each SES × protective factor combination, correcting separately for brain structural (12 comparisons, which included both global and system-level brain structure) and connectivity outcomes (11 comparisons including both global and system-level connectivity measures).

## 3 Results

### 3.1 Demographic information

See Table 1 for sample demographic information and descriptive statistics.

### 3.2 Sleep duration

#### 3.2.1 Income-to-needs ratio

**Global brain structure.** Sleep duration was found to significantly moderate the association between INR and both total brain volume and surface area, but not average cortical thickness (see Table 2 for significant model outputs and Table S6 for all model outputs). The positive association between INR and brain structure was weaker at higher levels of sleep duration (Fig. 1A–B). Simple slopes analysis revealed that, in low-income contexts, adolescents with longer sleep showed greater volume, surface area, and cortical thickness compared to those with shorter sleep (Table S9, Figure S7). Among high-income peers, such associations between sleep and brain structure were either absent or weaker.

**Table 2:** Statistics of significant interaction effects between SES and modifiable factors. The b values represent standardized b coefficients. Physical extracurricular activity variable winsorized at the 98th percentile. SE = standard error. df = degrees of freedom. Adj. p = false-discovery rate corrected p value.

*Predictor*	*Outcome*		*b*	*SE*	*df*	*t*	*p*	*Adj. p*
*Sleep Duration*								
ADI x Sleep	Average between-network rsFC	Global average	-0.027	0.008	8255	-3.22	0.001	0.014
		Somatosensory/motor	-0.026	0.010	8135	-2.63	0.009	0.047
INR x Sleep	Surface area	Total	-0.029	0.009	9937	-3.21	0.001	0.004
		Sensory	-0.030	0.009	9925	-3.36	0.001	0.003
		Somatosensory/motor	-0.024	0.009	9831	-2.64	0.008	0.012
		Higher-order association	-0.026	0.009	9550	-2.80	0.005	0.009
	Volume	Total	-0.031	0.009	9979	-3.43	0.001	0.003
		Sensory	-0.035	0.009	9940	-3.80	0.000	0.002
		Somatosensory/motor	-0.029	0.010	9836	-2.96	0.003	0.007
		Higher-order association	-0.027	0.009	9907	-2.89	0.004	0.008
	Average cortical thickness	Sensory	-0.023	0.010	9741	-2.31	0.021	0.028
		Somatosensory/motor	-0.022	0.010	9686	-2.17	0.030	0.036
	Average within-network rsFC	Higher-order association	-0.036	0.010	7986	-3.72	0.000	0.002
	Average between-network rsFC	Somatosensory/motor	0.030	0.011	7869	2.74	0.006	0.034
*Physical Extracurricular Activity (Winsorized)*								
INR x Physical extracurricular activity	Total surface area	Sensory	-0.024	0.009	9852	-2.77	0.006	0.034
	Total volume	Sensory	-0.027	0.009	9870	-3.09	0.002	0.024
*Non-Physical Extracurricular Activity*								
INR x Non-physical extracurricular activity	Total volume	Sensory	-0.026	0.008	10,047	-3.26	0.001	0.014

**Fig. 1:**
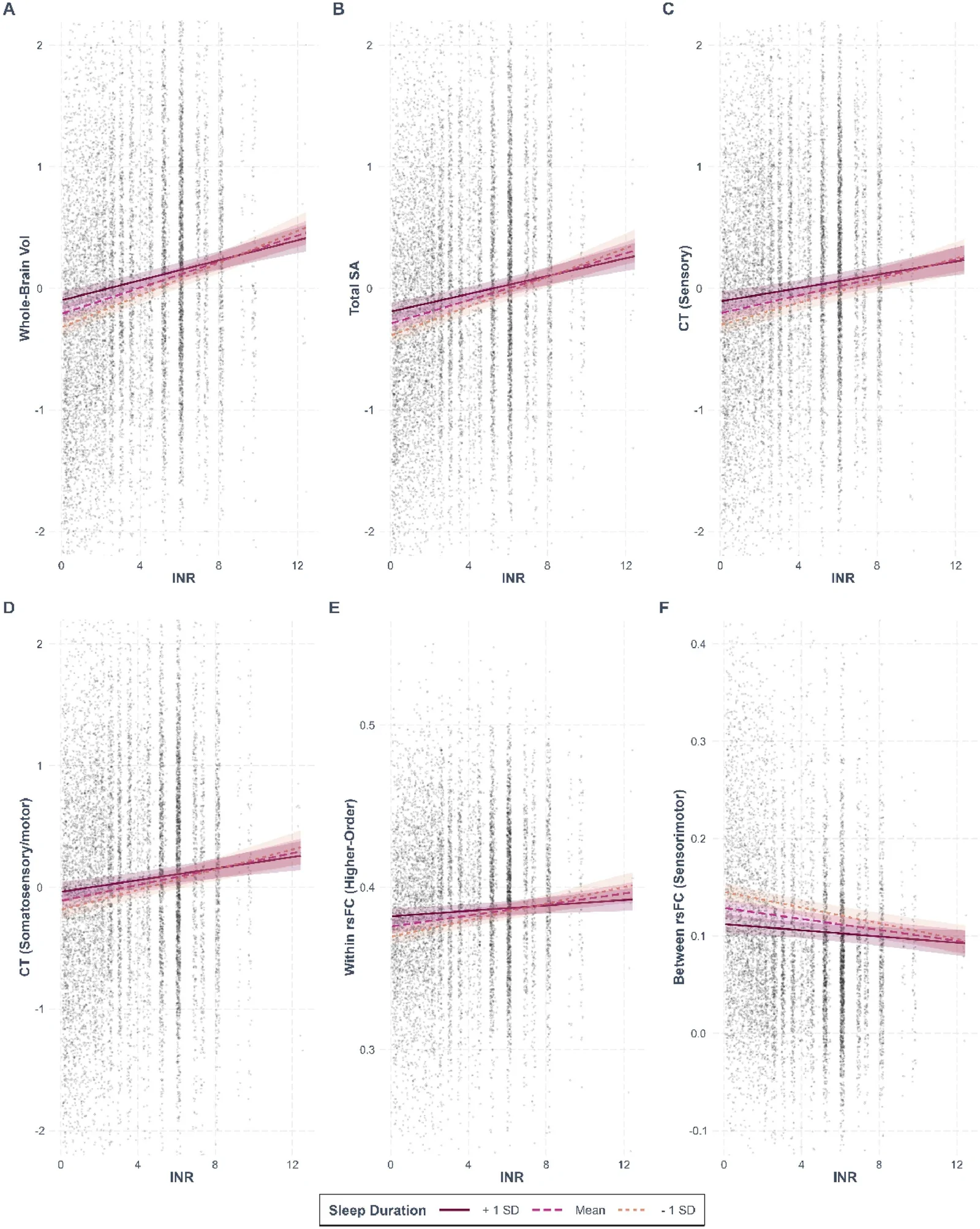
Moderating role of sleep duration in the association between income-to-needs ratio (INR) and brain measures. Panels A to D show associations between INR and brain structure at different sleep levels for: (A) total volume (vol), (B) total surface area (SA), (C) average cortical thickness (CT) of sensory cortices, (D) average CT of somatosensory/motor cortices. Panels E to F show associations between INR and resting-state functional connectivity (rsFC) at different levels of sleep for: (E) within-higher-order association network rsFC, and (F) between somatosensory/motor network rsFC. Volume and surface area (i.e. panel A and B) patterns were identical across the three systems, with system-specific figures available in the supplement (Fig. S3). For structural outcomes (Panels A-D), the y-axis shows standardized values; for rsFC measures (Panels E-F), the y-axis shows values in their original units for ease of interpretation. Slopes represent predicted values at mean ± 1 SD of sleep duration, and shaded areas indicate 95% confidence intervals. To enhance visibility of slopes, the y-axis scale has been reduced (see Fig. S11 for the full range) and a jitter of 0.1 has been added due to the high point density.

**System-level brain structure.** For volume and surface area, the patterns mirrored those for global brain metrics and were consistent across the three systems (i.e., sensory, somatosensory/motor and higher-order association systems). Although no significant interaction effects were observed for average cortical thickness at the global level, sleep duration significantly moderated associations between INR and thickness within sensory and somatosensory/motor systems (Table 2), with interaction patterns comparable to those observed for volume and surface area (Fig. 1C–D).

**Global resting-state functional connectivity.** Sleep duration was not found to significantly moderate the association between INR and average within-network and between-network connectivity globally (Table S6).

**System-level resting-state functional connectivity.** We found that the negative association between INR and average between-network rsFC of somatosensory/motor networks, as well as the positive association between INR and within-network connectivity of higher-order networks, was weaker at higher levels of sleep duration (Table 2, Fig. 1E–F). Simple slopes analyses indicated that the associations between sleep and rsFC differed by income context, with stronger associations observed at low income (Table S9, Figure S7).

#### 3.2.2 Neighborhood disadvantage

**Brain structure.** Associations between ADI and brain structure were not moderated by sleep duration (Table S6).

**Global resting-state functional connectivity.** Sleep duration significantly moderated the association between ADI and average between-network rsFC, revealing a complex pattern that depended on neighborhood context (Table 2). At high levels of neighborhood disadvantage, individuals with longer sleep duration showed lower between-network rsFC relative to those with shorter sleep duration, whereas the opposite pattern was observed at low levels of neighborhood disadvantage. Simple slopes analysis testing the association between sleep duration and rsFC at different levels of disadvantage showed that both the slopes for high ADI and low ADI were significant but in opposite directions (Table S9).

**System-level resting-state functional connectivity.** At the systems level, the negative association between ADI and average between-network rsFC of somatosensory/motor networks was weaker at longer sleep duration (Table 2, Fig. 2B). Simple slopes analysis revealed that sleep duration was more strongly associated with rsFC among low-income adolescents than among high-income peers (Table S9, Figure S8).

**Fig. 2:**
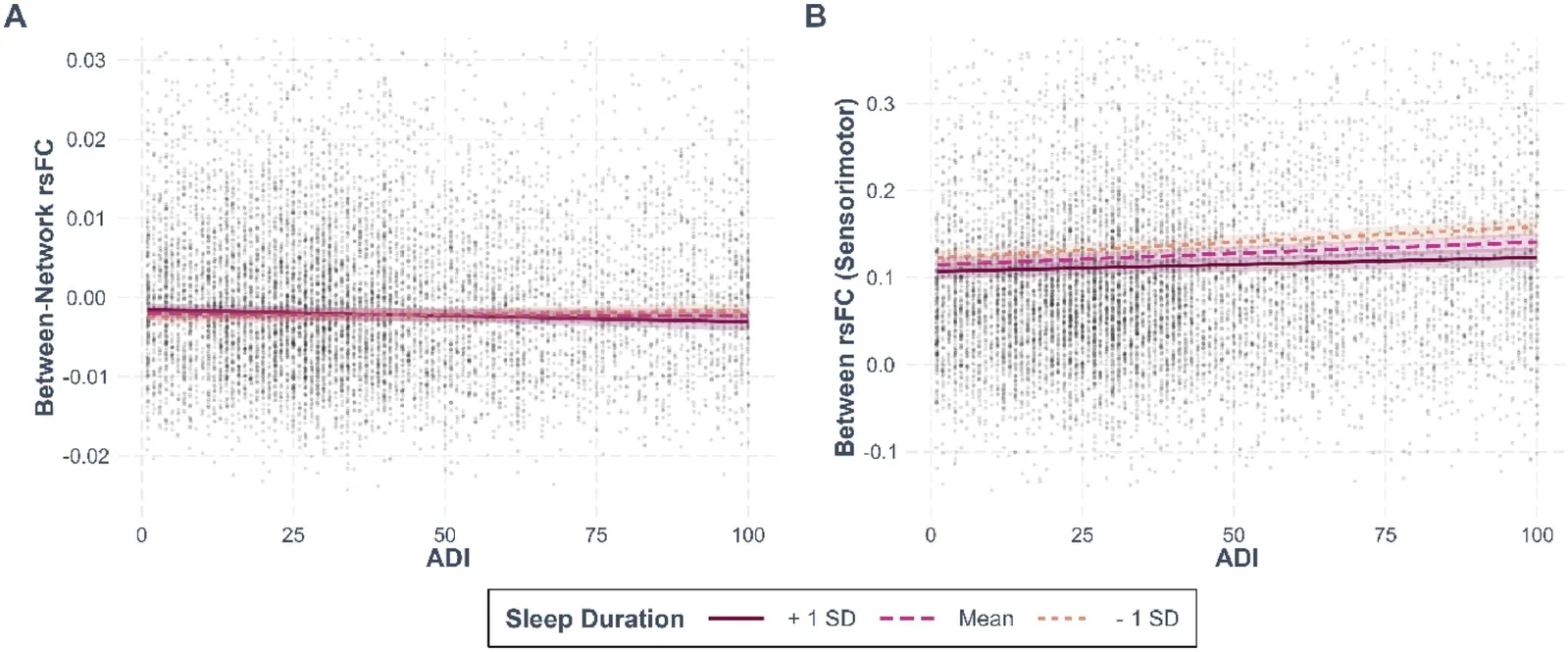
Association between area deprivation index (ADI) and resting-state functional connectivity (rsFC) at different levels of sleep for: (A) average between-network rsFC, and (B) average between-somatosensory/motor network rsFC. Slopes represent predicted values at mean ± 1 SD of sleep duration, and shaded areas indicate 95% confidence intervals. Higher levels of ADI indicate greater disadvantage. The y-axis shows rsFC values in their original units. To enhance visibility of slopes, the y-axis scale has been reduced (see Fig. S12 for the full range) and a jitter of 0.1 has been added due to the high point density.

### 3.3 Physical extracurricular activity

#### 3.3.1 Income-to-needs ratio

**Global brain structure.** Physical extracurricular activity was not found to moderate the associations between INR and global brain measures (Table S9).

**System-Level brain structure.** Physical extracurricular activity significantly moderated the associations between INR and both volume and surface area of sensory cortices. In both cases, physical activity reduced the strength of the association between INR and brain structure (Fig. 3). Simple slopes analyses indicated that adolescents from low-income backgrounds who had higher physical extracurricular activity showed greater volume and surface area of sensory regions than those with less physical activity, an effect that was absent in high-income contexts (Table S9, Figure S9).

**Fig. 3:**
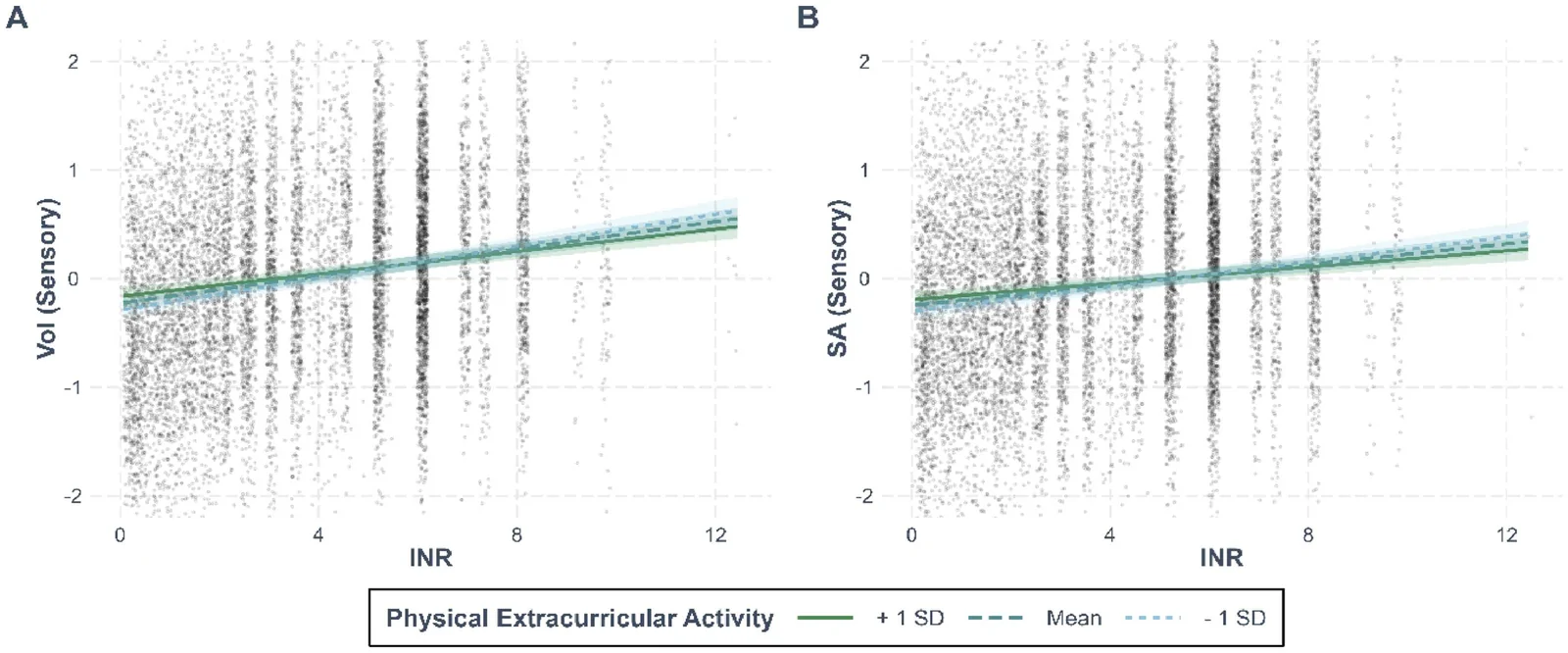
Association between income-to-needs ratio (INR) and brain structure at different levels of physical extracurricular activity for: (A) total volume (vol) of sensory cortices and (B) total surface area (SA) of sensory cortices. The y-axis shows standardized values. Slopes represent predicted values at mean ± 1 SD in physical extracurricular activity, and shaded areas indicate 95% confidence intervals. To enhance visibility of slopes, the y-axis scale has been reduced (see Fig. S13 for the full range) and a jitter of 0.1 has been added due to the high point density.

**Resting-state functional connectivity.** Associations between INR and within- or between-network rsFC were not moderated by engagement in physical extracurricular activity (Table S4).

#### 3.3.2 Neighborhood disadvantage

Engagement in physical extracurricular activity did not moderate associations between neighborhood disadvantage (ADI) and brain structure or rsFC (Table S4).

### 3.4 Non-physical extracurricular activity

#### 3.4.1 Income-to-needs ratio

**Global brain structure.** Associations between INR and global measures of brain structure were not moderated by non-physical extracurricular activity (Table S8).

**System-level brain structure.** Engagement in non-physical extracurricular activity moderated the association between INR and the volume of sensory cortices. Specifically, higher engagement was associated with a reduction in the strength of this positive association (Fig. 4). Simple slopes analysis revealed that low-income adolescents with greater participation in non-physical extracurriculars had greater sensory volume than peers with lower participation whereas no such effect was observed for high-income adolescents (Table S9, Figure S10).

**Fig. 4:**
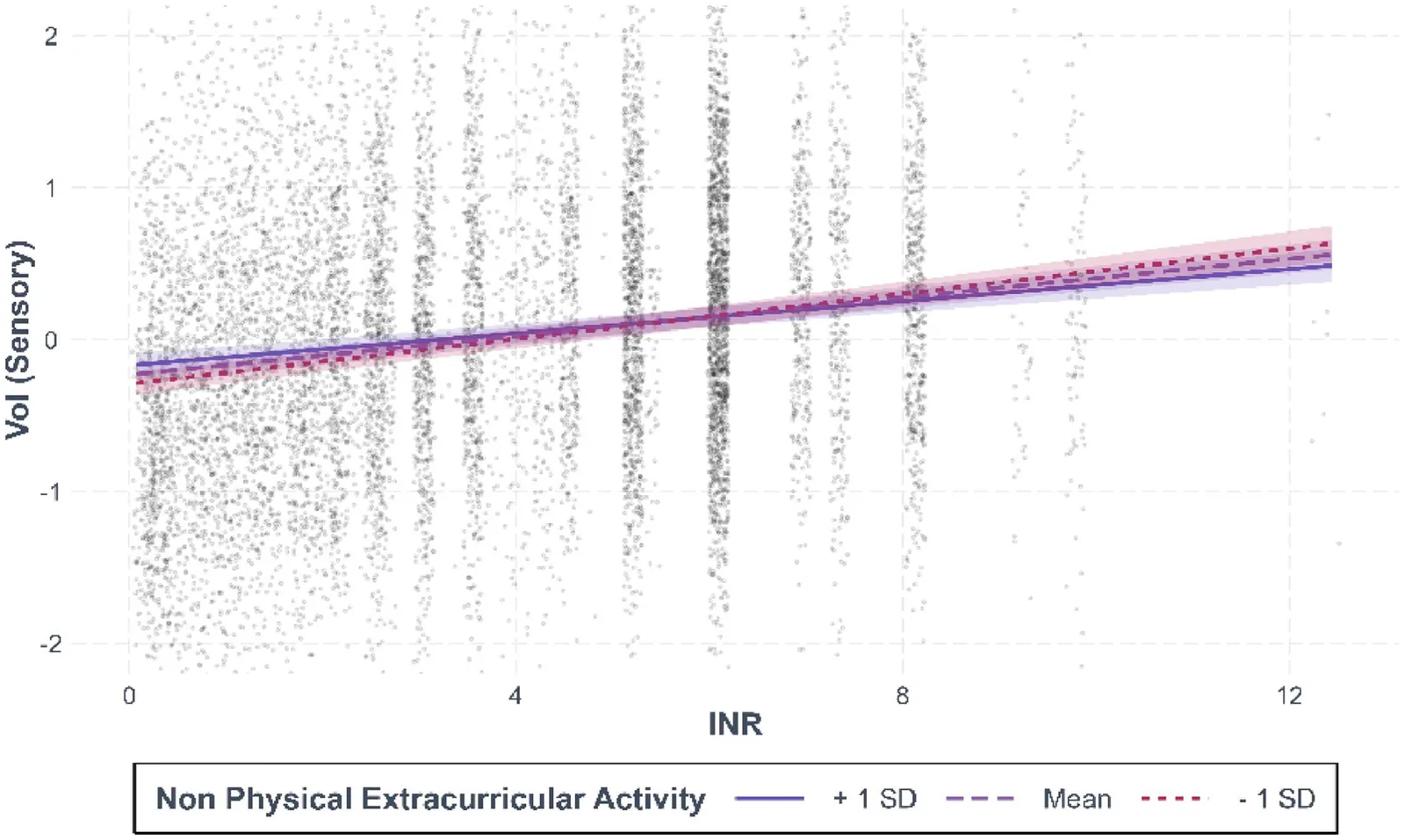
Association between income-to-needs ratio (INR) and total volume (vol) of sensory cortices at different levels of non-physical extracurricular activity. The y-axis shows standardized values. Slopes represent predicted values at mean ± 1 SD in physical extracurricular activity, and shaded areas indicate 95% confidence intervals. To enhance visibility of slopes, the y-axis scale has been reduced (see Fig. S14 for the full range) and a jitter of 0.1 has been added due to the high point density.

**Resting-state functional connectivity.** Non-physical extracurricular activity did not moderate associations between income-to-needs ratio and within- or between-network rsFC (Table S8).

#### 3.4.2 Neighborhood disadvantage

There was no evidence that engagement in non-physical extracurricular activities moderated associations between neighborhood disadvantage and brain outcomes (Table S8).

### 3.5 Sensitivity analyses with unwinsorized data, site, and a non-linear sleep term

When using the unwinsorized physical activity measure, findings for the volume of sensory cortices remained significant, whereas findings for surface area of sensory cortices did not (Table S3). When controlling for site, all results remained significant except for the interaction effect between sleep and INR for the thickness of somatosensory/motor cortices (Table S10). Finally, no interaction between the quadratic sleep term and SES reached significance (Table S11). However, our ability to detect such nonlinear effects may have been limited by the bounded nature of the sleep duration measure, which compresses variance at the upper end of the distribution.

## 4 Discussion

In this cross-sectional analysis of data from a large sample of early adolescents, we examined the moderating effects of modifiable lifestyle factors—including sleep duration and engagement in physical and non-physical extracurriculars—on associations between SES and both brain structure and rsFC. Higher sleep duration was associated with a weaker association between low SES and a range of brain measures—including surface area, volume, cortical thickness and functional connectivity. Additionally, associations between low income and the structure of sensory regions were weaker at higher levels of engagement in physical and non-physical extracurricular activities. Although effect sizes were small, even small effects may accumulate and have meaningful effects at the population level (Funder and Ozer, 2019). Nonetheless, their practical implications remain to be determined. Given that income- and neighborhood-related alterations in brain structure and connectivity are relevant for disparities in cognition and mental health (Rosen et al., 2025, Zhou et al., 2025), our findings identify these lifestyle factors, or the broader environmental contexts of which they may be markers, as important avenues for research on healthy child development in high-disadvantage contexts.

Consistent with our hypotheses, we found that longer sleep duration was associated with a reduction in the strength of the association between disadvantage—at both the household and neighborhood level—and global measures of brain structure and connectivity. Specifically, longer sleep duration was linked to weaker associations of income with global and system-specific surface area and volume, cortical thickness in sensory and somatosensory/motor regions, as well as rsFC between somatosensory/motor networks and within higher-order networks. It also moderated associations between neighborhood disadvantage and rsFC, namely global between-network connectivity and connectivity between somatosensory/motor networks. The observed associations should be considered within the broader ecological system in which sleep occurs. Sleep is embedded within interdependent family and neighborhood environments. Socioeconomic conditions may shape features of these environments—such as stressors and routines—that influence sleep, and sleep may in turn regulate the body’s physiological response to those same conditions (Grandner, 2019). Consistent with this, low sleep duration was less common among high SES youth in our sample (Figures S4 and S5). SES captures multiple aspects of a child’s household and neighborhood environment (Farah, 2017), including proximal stressors such as financial strain and harsh discipline, and broader contextual stressors (e.g., exposure to community violence) (Brown et al., 2019, Farah, 2017, Reiss et al., 2019). Converging evidence from animal models demonstrates that prolonged stress disrupts synaptic homeostasis and dendritic arborization, with downstream consequences for brain structure and functional connectivity (Eiland and Romeo, 2013, Hall et al., 2015, Liston and Gan, 2011). In these high-stress contexts, sleep may represent an important bio regulatory process through which physiological consequences of higher stress are regulated, essentially acting as a *restorative* process.

Sleep plays a crucial role in general brain health (Frank and Heller, 2019, Ibrahim et al., 2025), which could explain the widespread moderating effects observed in our study. It is important to note here that although our analyses focused on parent-reported sleep duration, sleep is more than simply its duration. Sleep health is a multidimensional construct encompassing duration, quality, timing, alertness and efficiency (Buysse, 2014). As such, the observed associations should be interpreted specifically in relation to sleep duration rather than sleep health more broadly. The glymphatic system—a metabolic waste clearing process in the brain that is most active during sleep—supports neural homeostasis by facilitating the clearance of metabolic waste and the delivery of nutrients (Bohr et al., 2022, Rasmussen et al., 2022). Animal studies further show that sleep plays a crucial role in synaptic plasticity (Sun et al., 2020), particularly synaptic turnover and pruning in adolescence (Maret et al., 2011). The moderating role of longer sleep duration for brain structure and function may therefore be highly salient during adolescence, a period marked by neurodevelopmental reorganization (Sanders et al., 2023, Stevens, 2016, Tamnes et al., 2017) and changes in sleep patterns (Colrain and Baker, 2011). However, because sleep is embedded within broader social environments (Grandner, 2019), the observed associations may also reflect unmeasured resources and environmental conditions that support longer sleep and brain development—including crowding, household organization, caregiver work schedules, and neighborhood noise and safety. Thus, these findings leave open whether the observed pattern is attributable to sleep itself, the broader environments of which sleep may be a marker, or both.

Previous work in the ABCD sample has shown that higher global volume and surface area are associated with lower mental health problems (Rakesh et al., 2025, Romer et al., 2023) and higher academic performance (Li et al., 2025). The observed associations between sleep duration and SES-related differences in brain structure may therefore have relevance for understanding variability in academic and mental health outcomes among youth from disadvantaged households and neighborhoods. The behavioral implications of the observed effects remain to be established. However, if our findings reflect the role of sleep rather than the broader environment, they would be broadly consistent with evidence suggesting that longer sleep moderates associations between low SES and lower cognitive function, higher internalizing and externalizing symptoms (Buckhalt et al., 2007, El-Sheikh et al., 2020, El-Sheikh et al., 2022), alongside intervention studies showing that increasing sleep duration is associated with improved cognitive outcomes in youth (Rey et al., 2020, Sadeh et al., 2003).

Although SES-brain links were generally weaker at higher levels of sleep duration, there was one exception for average between-network rsFC which showed a disordinal interaction pattern. Among disadvantaged youth, longer sleep was associated with lower average between-network rsFC, whereas among advantaged youth, longer sleep was associated with greater average between-network rsFC. This pattern must be interpreted with caution, however, given that directions of associations with between-network rsFC may vary across systems, and aggregation at the global level may blur these system-specific effects. For instance, system-specific analyses showed that connectivity between sensorimotor networks followed the expected pattern, with sleep associated with a weaker association between neighborhood disadvantage and higher connectivity. In the context of prior work in the ABCD sample showing lower connectivity between sensorimotor networks to be associated with higher cognitive scores and lower mental health problems (Modabbernia et al., 2021, Rakesh et al., 2021), a weaker association would be beneficial. However, as we did not examine associations with behavioral outcomes, it remains to be established whether these patterns are ultimately associated with adaptive or maladaptive outcomes—a direction for future work.

In addition to sleep, both physical and non-physical activity were associated with a similar reduction in the strength of associations among adolescents from low-income homes. Low-income contexts are suggested to be associated with lower cognitive stimulation (Rakesh, McLaughlin, et al., 2024), mediating associations between income and cognitive ability (Rakesh, Lee, et al., 2025). Converging evidence from animal and human studies links cognitive stimulation with dendritic morphology and cortical structure, suggesting that variation in stimulation is relevant to differences in cortical structure across income levels (Avants et al., 2015, Nithianantharajah and Hannan, 2006, Rosen et al., 2018). In this context, activities such as music training may represent one source of cognitive stimulation, given their task demands that integrate multiple sensory modalities, supporting plasticity (Herholz and Zatorre, 2012). Consistent with this, the moderating effects observed in this study were particularly relevant for the volume and area of sensory regions, including occipital visual and temporal cortices. It is therefore possible that the sensory processing demands of these activities engage sensory regions in ways that offset lower stimulation in low-income households. Theoretical models propose that disparities in cognitive stimulation—particularly early sensory input—shape the development of sensory processing regions, which, in turn, may shape prefrontal development (Rosen et al., 2019, Rosen et al., 2025). Following the sensory-to-association gradient of brain development (Larsen et al., 2023; D. J. Miller et al., 2012), income-related disparities in frontal regions may emerge later in development, scaffolded by earlier sensory disparities. In contrast, motor development effects may manifest earlier in life, potentially implicating factors more salient in infancy and toddlerhood such as play and tactile stimulation (Melo and Hoffman, 2024, Shibata et al., 2012, Trawick-Smith, 2014, Yogman et al., 2018). Longitudinal work spanning infancy through adolescence will be needed to evaluate these developmental pathways. It is important to note, however, that extracurricular participation is itself socially patterned (Snellman et al., 2015), with low participation being less common at high SES in our sample (Figures S4 and S5, Table S12). Low-SES youth who nonetheless engage in high levels of extracurricular activity may therefore differ from their lower-engagement peers not only in extracurricular participation, but in a broader set of unmeasured factors—such as parental investment, school quality, or residential stability—that co-occur with higher engagement. As such, the observed moderating effect may reflect these co-occurring advantages rather than a causal or independent effect of participation per se.

To the extent that the observed associations with brain outcomes extend to behavior, our findings in low-income contexts may align with some evidence showing that extracurricular engagement is linked to weaker associations between higher financial resources and cognitive, academic, and mental health outcomes (Heath et al., 2022, Kleszczewska et al., 2018, Pan et al., 2022, Rakesh et al., 2024), but contrast with studies reporting reinforced disparities (Bering and Schulz, 2024; Palou et al., 2024). However, the latter often rely on binary participation measures and narrow resource-intensive or school-based activities more accessible to youth from high income backgrounds, highlighting the need to capture diverse forms of participation. Complementing evidence that music interventions can improve mental health and resilience among low-income youth (Cheung et al., 2024), our results suggest that extracurricular engagement is a marker of variability in income-related differences in brain structure. In contrast, we found no such effects for functional connectivity measures. One potential explanation is the relatively state-dependent and variable nature of functional connectivity, as compared to structural measures that may more reliably reflect *cumulative* environmental influences. Alternatively, extracurricular activity may influence brain connectivity in more gradual experience-dependent pathways that manifest only later in development (Solé-Padullés et al., 2016), compared to the potentially more direct and immediate physiological effects of sleep on the brain (Ibrahim et al., 2025). More longitudinal work is needed to investigate these possibilities.

In contrast to our findings for income, and contrary to our hypotheses, extracurricular engagement did not moderate associations between neighborhood disadvantage and brain outcomes. Developmental work suggests that the associations between neighborhood disadvantage and the brain may depend on timing and youths’ degree of interaction with the neighborhood environment (Gard et al., 2021, Hyde et al., 2022). Building on Hyde et al. (2022), who emphasize that neighborhood influences are embedded within broader developmental and caregiving contexts, we speculate that the salience of neighborhood-level exposures may vary across adolescence as autonomy, peer engagement, and navigation of neighborhood spaces increase. Consequently, extracurricular engagement may become increasingly relevant as a moderating factor later in adolescence, when youth have greater direct exposure to neighborhood contexts (Hyde et al., 2022, Leventhal and Brooks-Gunn, 2000, Sampson et al., 2002) and more opportunities to benefit from structured activities, peer relationships, and community involvement. A related explanation is that different SES indicators may be associated with neurodevelopment through partially distinct pathways (Duncan and Magnuson, 2012). Household-level factors may be more strongly linked to opportunities for cognitive stimulation, whereas neighborhood disadvantage may be more closely associated with exposure to chronic environmental stressors. Such differences may help explain why extracurricular engagement showed moderating effects for income-related disadvantage but not neighborhood disadvantage in the present study, whereas sleep emerged as a marker of variability across both contexts. Longitudinal work spanning childhood through adolescence will be important for testing whether moderating mechanisms linked to neighborhood disadvantage shift across developmental stages.

This study has notable strengths, including its large sample size and the examination of both household and neighborhood SES. However, some limitations should be noted. One, although using baseline data allowed us to test associations with the largest possible sample, the cross-sectional design precludes establishing temporal ordering or causality. Because brain systems follow distinct developmental trajectories, cross-sectional analyses also cannot capture the full complexity of SES-lifestyle interactions across development or identify potential sensitive periods. Future research should thus leverage longitudinal designs spanning childhood and adolescence to characterize how these associations unfold over time. Two, sleep duration was measured via parent report using bounded categories rather than objective measures such as actigraphy. This results in compressed variability*—*particularly at the upper end of the distribution (9–11 h) where many adolescents fall (mean of approximately 9 h in our sample)*—*which limits interpretation of associations at higher levels of sleep duration. Moreover, prior work suggests older adolescent self-reports overestimate sleep duration relative to actigraphy by approximately 28 min, and that this overestimation may be greater among lower-SES youth (White et al., 2026). Because this error is patterned by SES rather than random, standard assumptions about attenuation toward the null may not apply. Future work could leverage the actigraphy subsample available in later ABCD waves to evaluate whether the present SES × sleep interactions are robust to this reporting bias. Additionally, because sleep health is multidimensional— encompassing duration, timing, regularity, and quality—our sleep duration variable captures only one aspect of sleep health. Future studies using actigraphy-based measures will provide a more comprehensive characterization of sleep. Three, extracurricular activities were heterogeneous; for example, non-physical activity included arts, crafts (e.g. knitting), games (e.g. chess), drama, and musical instrument playing. Future work should examine these activities separately to determine which activities may promote resilience in low-income contexts. Four, although the aim of the study is to characterize factors that may explain heterogeneity in neural outcomes among youth from lower SES backgrounds, it is important to acknowledge that some of this heterogeneity may also reflect measurement error of SES, as single proxy indicators such as income cannot fully capture the multidimensional nature of socioeconomic disadvantage. Five, our moderators are socially patterned (Bobba et al., 2023, Fronberg et al., 2022, Snellman et al., 2015), and high levels of these factors were less common among youth from low SES backgrounds in our sample (Figures S4 and S5, Table S12). The present findings should therefore not be interpreted as independent effects of sleep duration or extracurricular engagement, as they may also reflect unmeasured resources and environmental conditions associated with these behaviors in low-SES contexts. Six, main effects were not investigated but modeled as linear. If a main effect is curvilinear, the linear specification could absorb some of the unmodeled curvature and inflate the interaction effect. This is particularly relevant for sleep, which often shows non-linear associations with developmental outcomes (James and Hale, 2017). Seven, participants excluded due to failed MRI quality control or missing scans differed systematically from the analytic sample, which may limit the generalizability of findings. Eight, the ability to reliably determine the practical importance or cumulative consequences of small effects remains underdeveloped (Sauer and Drummond, 2020). Finally, studies employing moderated mediation frameworks are needed to determine whether these effects translate into meaningful behavioral outcomes. It is also important to note that these differences may be adaptive for certain behavioral outcomes (DeJoseph et al., 2024, Ellis et al., 2017, Ellwood-Lowe et al., 2021). Future work should therefore integrate deficit and strength-based approaches by including measures of behavioral and cognitive skills relevant to the contexts disadvantaged youth typically navigate (e.g., attention-shifting in high-stress contexts).

Altogether, the findings of the current study suggest that longer sleep and greater extracurricular involvement may help reduce the strength of the association between SES and brain structure and function, with more pronounced effects among individuals from low-income backgrounds. Future longitudinal and intervention research should evaluate whether these factors can actively reduce SES disparities, investigate the mechanisms underlying these associations, and determine whether SES–lifestyle interactions extend to behavioral outcomes.

## Data statement

The data used in this project are publicly available (https://abcdstudy.org/). Access to the data is granted to qualified researchers via a data use agreement. For further information on how to obtain access to this dataset, visit the NIH Brain Development Cohorts data sharing platform (https://www.nbdc-datahub.org/).

## CRediT authorship contribution statement

**Juliette Tinch:** Writing – review & editing, Writing – original draft, Visualization, Methodology, Investigation, Formal analysis, Data curation, Conceptualization. **Phoebe Thomson:** Writing – review & editing, Writing – original draft, Supervision, Methodology, Conceptualization. **Divyangana Rakesh:** Writing – review & editing, Writing – original draft, Supervision, Methodology, Funding acquisition, Conceptualization.

## Declaration of Competing Interest

The authors declare that they have no known competing financial interests or personal relationships that could have appeared to influence the work reported in this paper.

## Data Availability

The authors do not have permission to share data.

## References

[bib1] Albakri U., Drotos E., Meertens R. (2021). Sleep Health Promotion Interventions and Their Effectiveness: An Umbrella Review. Int. J. Environ. Res. Public. Health.

[bib2] Astill R.G., Van der Heijden K.B., Van Ijzendoorn M.H., Van Someren E.J.W. (2012). Sleep, cognition, and behavioral problems in school-age children: A century of research meta-analyzed. Psychol. Bull..

[bib3] Avants B.B., Hackman D.A., Betancourt L.M., Lawson G.M., Hurt H., Farah M.J. (2015). Relation of Childhood Home Environment to Cortical Thickness in Late Adolescence: Specificity of Experience and Timing. PLOS One.

[bib4] Benjamini Y., Hochberg Y. (1995). Controlling the False Discovery Rate: A Practical and Powerful Approach to Multiple Testing. J. R. Stat. Soc. Ser. B Stat. Methodol..

[bib5] Bering H., Schulz W. (2024). Can sports and music level the playing field? Adolescents’ extracurricular activities and the reproduction of social inequalities in cognitive skills. Res. Soc. Stratif. Mobil..

[bib6] Biddle S.J.H., Ciaccioni S., Thomas G., Vergeer I. (2019). Physical activity and mental health in children and adolescents: An updated review of reviews and an analysis of causality. Psychol. Sport. Exerc..

[bib7] Boardman J.D., Saint Onge J.M. (2005). Neighborhoods and Adolescent Development. Child. Youth Environ..

[bib8] Bobba B., Bacaro V., Crocetti E. (2023). Embedded in Contexts: A Systematic Review of the Longitudinal Associations Between Contextual Factors and Sleep. Adolesc. Res. Rev..

[bib9] Boelens M., Smit M.S., Raat H., Bramer W.M., Jansen W. (2022). Impact of organized activities on mental health in children and adolescents: An umbrella review. Prev. Med. Rep..

[bib10] Bohr T., Hjorth P.G., Holst S.C., Hrabětová S., Kiviniemi V., Lilius T., Lundgaard I., Mardal K.-A., Martens E.A., Mori Y., Nägerl U.V., Nicholson C., Tannenbaum A., Thomas J.H., Tithof J., Benveniste H., Iliff J.J., Kelley D.H., Nedergaard M. (2022). The glymphatic system: Current understanding and modeling. iScience.

[bib11] Bottenhorn K.L., Cardenas-Iniguez C., Schachner J.N., Rosario M.A., Mills K.L., Laird A.R., Herting M.M. (2024). Adolescent Neurodevelopmental Variance Across Social Strata. JAMA Netw. Open..

[bib12] Brito N.H., Noble K.G., & the Pediatric Imaging, G. S., Neurocognition (2018). The independent and interacting effects of socioeconomic status and dual-language use on brain structure and cognition. Dev. Sci..

[bib13] Brody G.H., Yu T., Nusslock R., Barton A.W., Miller G.E., Chen E., Holmes C., McCormick M., Sweet L.H. (2019). The Protective Effects of Supportive Parenting on the Relationship Between Adolescent Poverty and Resting-State Functional Brain Connectivity During Adulthood. Psychol. Sci..

[bib14] Brown E.D., Anderson K.E., Garnett M.L., Hill E.M. (2019). Economic instability and household chaos relate to cortisol for children in poverty. J. Fam. Psychol..

[bib15] Bruni O., Ottaviano S., Guidetti V., Romoli M., Innocenzi M., Cortesi F., Giannotti F. (1996). The Sleep Disturbance Scale for Children (SDSC). Construction and validation of an instrument to evaluate sleep disturbances in childhood and adolescence. J. Sleep. Res..

[bib16] Buckhalt J.A., El-Sheikh M., Keller P. (2007). Children’s Sleep and Cognitive Functioning: Race and Socioeconomic Status as Moderators of Effects. Child. Dev..

[bib17] Buysse D.J. (2014). Sleep Health: Can We Define It? Does It Matter?. Sleep.

[bib18] Cabane C., Hille A., Lechner M. (2016). Mozart or Pelé? The effects of adolescents' participation in music and sports. Labour Econ..

[bib19] Carrick C., Rakesh D., Michel L., Bates K., Fuhrmann D. (2026). Individual differences in adolescent cortical development are associated with neighborhood characteristics: Longitudinal findings from the ABCD study. Cereb. Cortex.

[bib20] Carroll S.L., Shewark E.A., Mikhail M.E., Thaler D.J., Pearson A.L., Klump K.L., Burt S.A. (2024). Identifying the ‘active ingredients’ of socioeconomic disadvantage for youth outcomes in middle childhood. Dev. Psychopathol..

[bib21] Cheung A.T., Ho L.L.K., Li W.H.C., Chan G.C.F., Choi K.C., Chung J.O.K., Chan C.Y.W.H. (2024). Group-based instrumental musical training to enhance resilience among school-aged children from low-income families: A pilot randomised waitlist controlled trial. Nurs. Open..

[bib22] Colrain I.M., Baker F.C. (2011). Changes in sleep as a function of adolescent development. Neuropsychol. Rev..

[bib23] Crawford D., Timperio A., Giles-Corti B., Ball K., Hume C., Roberts R., Andrianopoulos N., Salmon J. (2008). Do features of public open spaces vary according to neighbourhood socio-economic status?. Health Place..

[bib24] de Greeff J.W., Bosker R.J., Oosterlaan J., Visscher C., Hartman E. (2018). Effects of physical activity on executive functions, attention and academic performance in preadolescent children: A meta-analysis. J. Sci. Med. Sport..

[bib25] DeJoseph M.L., Ellwood-Lowe M.E., Miller-Cotto D., Silverman D., Shannon K.A., Reyes G., Rakesh D., Frankenhuis W.E. (2024). The promise and pitfalls of a strength-based approach to child poverty and neurocognitive development: Implications for policy. Dev. Cogn. Neurosci..

[bib26] Desikan R.S., Ségonne F., Fischl B., Quinn B.T., Dickerson B.C., Blacker D., Buckner R.L., Dale A.M., Maguire R.P., Hyman B.T., Albert M.S., Killiany R.J. (2006). An automated labeling system for subdividing the human cerebral cortex on MRI scans into gyral based regions of interest. NeuroImage.

[bib27] Duncan G.J., Magnuson K. (2012). Socioeconomic status and cognitive functioning: Moving from correlation to causation. WIREs Cogn. Sci..

[bib28] Duncan G.J., Magnuson K.A. (2003). Socioeconomic status, parenting, and child development.

[bib29] Duncan G.J., Morris P.A., Rodrigues C. (2011). Does money really matter? Estimating impacts of family income on young children’s achievement with data from random-assignment experiments. Dev. Psychol..

[bib30] Eiland L., Romeo R.D. (2013). Stress and the developing adolescent brain. Neurosci. Stress. Adolesc. Brain.

[bib31] El-Sheikh M., Gillis B.T., Saini E.K., Erath S.A., Buckhalt J.A. (2022). Sleep and disparities in child and adolescent development. Child. Dev. Perspect..

[bib32] El-Sheikh M., Shimizu M., Philbrook L.E., Erath S.A., Buckhalt J.A. (2020). Sleep and development in adolescence in the context of socioeconomic disadvantage. J. Adolesc..

[bib33] Ellis B.J., Bianchi J., Griskevicius V., Frankenhuis W.E. (2017). Beyond risk and protective factors: an adaptation-based approach to resilience. Perspect. Psychol. Sci..

[bib34] Ellwood-Lowe M.E., Whitfield-Gabrieli S., Bunge S.A. (2021). Brain network coupling associated with cognitive performance varies as a function of a child’s environment in the ABCD study. Nat. Commun..

[bib35] Ernst M., Torrisi S., Balderston N., Grillon C., Hale E.A. (2015). fMRI Functional Connectivity Applied to Adolescent Neurodevelopment*. Annu. Rev. Clin. Psychol..

[bib36] Evans G.W. (2004). The Environment of Childhood Poverty. Am. Psychol..

[bib37] Farah M.J. (2017). The Neuroscience of Socioeconomic Status: Correlates, Causes, and Consequences. Neuron.

[bib38] Farah M.J. (2018). Socioeconomic status and the brain: Prospects for neuroscience-informed policy. Nat. Rev. Neurosci..

[bib39] Ferreira V.R., Carvalho L.B.C., Ruotolo F., de Morais J.F., Prado L.B.F., Prado G.F. (2009). Sleep disturbance scale for children: Translation, cultural adaptation, and validation. Sleep. Med..

[bib40] Frank M.G., Heller H.C. (2019). The Function(s) of Sleep. Handb. Exp. Pharmacol..

[bib41] Franzini L., Taylor W., Elliott M.N., Cuccaro P., Tortolero S.R., Janice Gilliland M., Grunbaum J., Schuster M.A. (2010). Neighborhood characteristics favorable to outdoor physical activity: Disparities by socioeconomic and racial/ethnic composition. Health Place..

[bib42] Fronberg K.M., Bai S., Teti D.M. (2022). Household chaos mediates the link between family resources and child sleep. Sleep. Health.

[bib43] Funder D.C., Ozer D.J. (2019). Evaluating Effect Size in Psychological Research: Sense and Nonsense. Adv. Methods Pract. Psychol. Sci..

[bib44] Garavan H., Bartsch H., Conway K., Decastro A., Goldstein R.Z., Heeringa S., Jernigan T., Potter A., Thompson W., Zahs D. (2018). Recruiting the ABCD sample: Design considerations and procedures. Dev. Cogn. Neurosci..

[bib45] Gard A.M., Maxwell A.M., Shaw D.S., Mitchell C., Brooks-Gunn J., McLanahan S.S., Forbes E.E., Monk C.S., Hyde L.W. (2021). Beyond family-level adversities: Exploring the developmental timing of neighborhood disadvantage effects on the brain. Dev. Sci..

[bib46] Gordon E.M., Laumann T.O., Adeyemo B., Huckins J.F., Kelley W.M., Petersen S.E. (2014). Generation and Evaluation of a Cortical Area Parcellation from Resting-State Correlations. Cerebral Cortex.

[bib47] Grandner M.A., Grandner M.A. (2019). Sleep and Health.

[bib48] Hackman D.A., Cserbik D., Chen J.-C.C., Berhane K., Minaravesh B., McConnell R., Herting M.M. (2021). Association of local variation in neighborhood disadvantage in metropolitan areas with youth neurocognition and brain structure. JAMA Pediatr..

[bib49] Hackman D.A., Gallop R., Evans G.W., Farah M.J. (2015). Socioeconomic status and executive function: Developmental trajectories and mediation. Dev. Sci..

[bib50] Hagler D.J., Hatton S.N., Cornejo M.D., Makowski C., Fair D.A., Dick A.S., Sutherland M.T., Casey B.J., Barch D.M., Harms M.P., Watts R., Bjork J.M., Garavan H.P., Hilmer L., Pung C.J., Sicat C.S., Kuperman J., Bartsch H., Xue F., Heitzeg M.M., Laird A.R., Trinh T.T., Gonzalez R., Tapert S.F., Riedel M.C., Squeglia L.M., Hyde L.W., Rosenberg M.D., Earl E.A., Howlett K.D., Baker F.C., Soules M., Diaz J., de Leon O.R., Thompson W.K., Neale M.C., Herting M., Sowell E.R., Alvarez R.P., Hawes S.W., Sanchez M., Bodurka J., Breslin F.J., Morris A.S., Paulus M.P., Simmons W.K., Polimeni J.R., van der Kouwe A., Nencka A.S., Gray K.M., Pierpaoli C., Matochik J.A., Noronha A., Aklin W.M., Conway K., Glantz M., Hoffman E., Little R., Lopez M., Pariyadath V., Weiss S.R.B., Wolff-Hughes D.L., DelCarmen-Wiggins R., Feldstein Ewing S.W., Miranda-Dominguez O., Nagel B.J., Perrone A.J., Sturgeon D.T., Goldstone A., Pfefferbaum A., Pohl K.M., Prouty D., Uban K., Bookheimer S.Y., Dapretto M., Galvan A., Bagot K., Giedd J., Infante M.A., Jacobus J., Patrick K., Shilling P.D., Makowski R., Li Y., Sugrue L., Banich M.T., Friedman N., Hewitt J.K., Hopfer C., Sakai J., Tanabe J., Cottler L.B., Nixon S.J., Chang L., Cloak C., Ernst T., Reeves G., Kennedy D.N., Heeringa S., Peltier S., Schulenberg J., Sripada C., Zucker R.A., Iacono W.G., Luciana M., Calabro F.J., Clark D.B., Lewis D.A., Luna B., Schirda C., Brima T., Foxe J.J., Freedman E.G., Mruzek D.W., Mason M.J., Huber R., McGlade E., Prescot A., Renshaw P.F., Yurgelun-Todd K.M., Allgaier N.A., Dumas J.A., Ivanova M., Potter A., Florsheim P., Larson C., Lisdahl K., Charness M.E., Fuemmeler B., Hettema J.M., Maes H.H., Steinberg J., Anokhin A.P., Glaser P., Heath A.C., Madden P.A., Baskin-Sommers A., Constable R.T., Grant S.J., Dowling G.J., Brown S.A., Jernigan T.L., Dale A.M. (2019). Image processing and analysis methods for the Adolescent Brain Cognitive Development Study. NeuroImage.

[bib51] Hall B.S., Moda R.N., Liston C. (2015). Glucocorticoid mechanisms of functional connectivity changes in stress-related neuropsychiatric disorders. Neurobiol. Stress, Stress Resil..

[bib52] Hanson J.L., Hair N., Shen D.G., Shi F., Gilmore J.H., Wolfe B.L., Pollak S.D. (2013). Family poverty affects the rate of human infant brain growth. PLOS One.

[bib53] Heath R.D., Anderson C., Turner A.C., Payne C.M. (2022). Extracurricular activities and disadvantaged youth: a complicated—but promising—story. Urban. Educ..

[bib54] Herholz S.C., Zatorre R.J. (2012). Musical training as a framework for brain plasticity: behavior, function, and structure. Neuron.

[bib55] Hosker D.K., Elkins R.M., Potter M.P. (2019). Promoting mental health and wellness in youth through physical activity, nutrition, and sleep. Child. Adolesc. Psychiatr. Clin..

[bib56] Huppertz C., Bartels M., de Zeeuw E.L., van Beijsterveldt C.E.M., Hudziak J.J., Willemsen G., Boomsma D.I., de Geus E.J.C. (2016). Individual Differences in Exercise Behavior: Stability and Change in Genetic and Environmental Determinants From Age 7 to 18. Behav. Genet..

[bib57] Hyde L.W., Gard A.M., Tomlinson R.C., Suarez G.L., Westerman H.B. (2022). Parents, neighborhoods, and the developing brain. Child. Dev. Perspect..

[bib58] Ibrahim A., Högl B., Stefani A. (2025). Sleep as the foundation of brain health. Semin. Neurol..

[bib59] Ishiguro C., Ishihara T., Morita N. (2023). Extracurricular music and visual arts activities are related to academic performance improvement in school-aged children. NPJ Sci. Learn..

[bib60] Jago R., Sebire S.J., Davies B., Wood L., Banfield K., Edwards M.J., Powell J.E., Montgomery A.A., Thompson J.L., Fox K.R. (2015). Increasing children’s physical activity through a teaching-assistant led extracurricular intervention: Process evaluation of the action 3:30 randomised feasibility trial. BMC Public Health.

[bib61] James S., Hale L. (2017). Sleep duration and child well-being: a nonlinear association. J. Clin. Child. Adolesc. Psychol..

[bib62] Judd N., Sauce B., Wiedenhoeft J., Tromp J., Chaarani B., Schliep A., van Noort B., Penttilä J., Grimmer Y., Insensee C., Becker A., Banaschewski T., Bokde A.L.W., Quinlan E.B., Desrivières S., Flor H., Grigis A., Gowland P., Heinz A., Klingberg T. (2020). Cognitive and brain development is independently influenced by socioeconomic status and polygenic scores for educational attainment. Proc. Natl. Acad. Sci..

[bib63] Kleszczewska D., Dzielska A., Salonna F., Mazur J. (2018). The association between physical activity and general life satisfaction in lower secondary school students: the role of individual and family factors. Community Ment. Health J..

[bib64] Knussmann G.N., Anderson J.S., Prigge M.B.D., Dean D.C., Lange N., Bigler E.D., Alexander A.L., Lainhart J.E., Zielinski B.A., King J.B. (2022). Test-retest reliability of FreeSurfer-derived volume, area and cortical thickness from MPRAGE and MP2RAGE brain MRI images. Neuroimage: Reports.

[bib65] Larsen B., Sydnor V.J., Keller A.S., Yeo B.T.T., Satterthwaite T.D. (2023). A critical period plasticity framework for the sensorimotor–association axis of cortical neurodevelopment. Trends Neurosci..

[bib66] Leventhal T., Brooks-Gunn J. (2000). The neighborhoods they live in: The effects of neighborhood residence on child and adolescent outcomes. Psychol. Bull..

[bib67] Li Q., Whittle S., Rakesh D. (2025). Longitudinal associations between greenspace exposure, structural brain development, and mental health and academic performance during early adolescence. Biol. Psychiatry.

[bib68] Liston C., Gan W.-B. (2011). Glucocorticoids are critical regulators of dendritic spine development and plasticity in vivo. Proc. Natl. Acad. Sci..

[bib69] Luby J., Belden A., Botteron K., Marrus N., Harms M.P., Babb C., Nishino T., Barch D. (2013). The effects of poverty on childhood brain development: the mediating effect of caregiving and stressful life events. JAMA Pediatr..

[bib70] Mackey A.P., Finn A.S., Leonard J.A., Jacoby-Senghor D.S., West M.R., Gabrieli C.F.O., Gabrieli J.D.E. (2015). Neuroanatomical Correlates of the Income-Achievement Gap. Psychol. Sci..

[bib71] Maret S., Faraguna U., Nelson A.B., Cirelli C., Tononi G. (2011). Sleep and wake modulate spine turnover in the adolescent mouse cortex. Nat. Neurosci..

[bib72] Marquet O., Hipp J.A., Alberico C., Huang J.-H., Fry D., Mazak E., Lovasi G.S., Floyd M.F. (2019). Short-term associations between objective crime, park-use, and park-based physical activity in low-income neighborhoods. Prev. Med..

[bib73] McDermott C.L., Seidlitz J., Nadig A., Liu S., Clasen L.S., Blumenthal J.D., Reardon P.K., Lalonde F., Greenstein D., Patel R., Chakravarty M.M., Lerch J.P., Raznahan A. (2019). Longitudinally mapping childhood socioeconomic status associations with cortical and subcortical morphology. J. Neurosci..

[bib74] McKenzie T.L., Moody J.S., Carlson J.A., Lopez N.V., Elder J.P. (2013). Neighborhood Income Matters: Disparities in Community Recreation Facilities, Amenities, and Programs. J. Park. Recreat. Adm..

[bib75] Melo A.I., Hoffman K.L., Rezaei N., Yazdanpanah N. (2024). PsychoNeuroImmunology: Volume 1: Integration of Psychology, Neurology, and Immunology.

[bib76] Melzer T.R., Keenan R.J., Leeper G.J., Kingston-Smith S., Felton S.A., Green S.K., Henderson K.J., Palmer N.J., Shoorangiz R., Almuqbel M.M., Myall D.J. (2020). Test-retest reliability and sample size estimates after MRI scanner relocation. NeuroImage.

[bib77] Merz E.C., Strack J., Hurtado H., Vainik U., Thomas M., Evans A., Khundrakpam B. (2022). Educational attainment polygenic scores, socioeconomic factors, and cortical structure in children and adolescents. Human. Brain Mapp..

[bib78] Michael C., Taxali A., Angstadt M., Kardan O., Weigard A., Molloy M.F., McCurry K.L., Hyde L.W., Heitzeg M.M., Sripada C. (2024). Socioeconomic resources in youth are linked to divergent patterns of network integration/segregation across the brain’s transmodal axis. PNAS Nexus.

[bib79] Michael C., Tillem S., Sripada C.S., Burt S.A., Klump K.L., Hyde L.W. (2023). Neighborhood poverty during childhood prospectively predicts adolescent functional brain network architecture. Dev. Cogn. Neurosci..

[bib80] Miller D.J., Duka T., Stimpson C.D., Schapiro S.J., Baze W.B., McArthur M.J., Fobbs A.J., Sousa A.M.M., Šestan N., Wildman D.E., Lipovich L., Kuzawa C.W., Hof P.R., Sherwood C.C. (2012). Prolonged myelination in human neocortical evolution. Proc. Natl. Acad. Sci..

[bib81] Miller J.G., López V., Buthmann J.L., Garcia J.M., Gotlib I.H. (2022). A Social gradient of cortical thickness in adolescence: relationships with neighborhood socioeconomic disadvantage, family socioeconomic status, and depressive symptoms. Biol. Psychiatry Glob. Open Sci..

[bib82] Modabbernia A., Janiri D., Doucet G.E., Reichenberg A., Frangou S. (2021). Multivariate patterns of brain-behavior-environment associations in the adolescent brain and cognitive development study. Biol. Psychiatry Nov. Neurodev. Disturb. Autism Risk.

[bib83] Morris D.S. (2015). Actively closing the gap? Social class, organized activities, and academic achievement in high school. Youth Soc..

[bib84] Musshafen L.A., Tyrone R.S., Abdelaziz A., Sims-Gomillia C.E., Pongetti L.S., Teng F., Fletcher L.M., Reneker J.C. (2021). Associations between sleep and academic performance in US adolescents: A systematic review and meta-analysis. Sleep. Med..

[bib85] National Center for Education Statistics (2012). Improving the Measurement of Socioeconomic Status for the National Assessment of Educational Progress: A Theoretical Foundation--Recommendations to the National Center for Education Statistics. Natl. Cent. Educ. Stat..

[bib86] Nithianantharajah J., Hannan A.J. (2006). Enriched environments, experience-dependent plasticity and disorders of the nervous system. Nat. Rev. Neurosci..

[bib87] Noble K.G., Giebler M.A. (2020). The neuroscience of socioeconomic inequality. Curr. Opin. Behav. Sci. Sensitive Crit. Periods.

[bib88] Noble K.G., Houston S.M., Brito N.H., Bartsch H., Kan E., Kuperman J.M., Akshoomoff N., Amaral D.G., Bloss C.S., Libiger O., Schork N.J., Murray S.S., Casey B.J., Chang L., Ernst T.M., Frazier J.A., Gruen J.R., Kennedy D.N., Van Zijl P., Sowell E.R. (2015). Family income, parental education and brain structure in children and adolescents. Nat. Neurosci..

[bib89] Noble K.G., McCandliss B.D., Farah M.J. (2007). Socioeconomic gradients predict individual differences in neurocognitive abilities. Dev. Sci..

[bib90] Norbom L.B., Ferschmann L., Parker N., Agartz I., Andreassen O.A., Paus T., Westlye L.T., Tamnes C.K. (2021). New insights into the dynamic development of the cerebral cortex in childhood and adolescence: Integrating macro- and microstructural MRI findings. Progress. Neurobiol..

[bib91] Norbom L.B., Rokicki J., Eilertsen E.M., Wiker T., Hanson J., Dahl A., Alnæs D., Fernández-Cabello S., Beck D., Agartz I., Andreassen O.A., Westlye L.T., Tamnes C.K. (2024). Parental education and income are linked to offspring cortical brain structure and psychopathology at 9–11 years. JCPP Adv..

[bib92] Palou A., Ursin P.A., Demanet J. (2024). Social inequality in skills: Exploring the moderating role of extracurricular activities related to socio‐economic differences in non‐cognitive and cognitive outcomes. Eur. J. Educ..

[bib93] Pan Y., Zhou D., Shek D.T.L. (2022). Participation in AFter-school Extracurricular Activities and Cognitive Ability among Early Adolescents in China: Moderating Effects of Gender and Family Economic Status. Front. Pediatr..

[bib94] Pascoe M., Bailey A.P., Craike M., Carter T., Patten R., Stepto N., Parker A. (2020). Physical activity and exercise in youth mental health promotion: A scoping review. BMJ Open. Sport. & Exerc. Med..

[bib95] Philbrook L.E., Saini E.K., Fuller-Rowell T.E., Buckhalt J.A., El-Sheikh M. (2020). Socioeconomic status and sleep in adolescence: The role of family chaos. J. Fam. Psychol..

[bib96] Rakesh D., Cropley V., Zalesky A., Vijayakumar N., Allen N.B., Whittle S. (2021). Neighborhood disadvantage and longitudinal brain-predicted-age trajectory during adolescence. Dev. Cogn. Neurosci..

[bib97] Rakesh D., Lee P.A., Gaikwad A., McLaughlin K.A. (2025). Annual Research Review: Associations of socioeconomic status with cognitive function, language ability, and academic achievement in youth: a systematic review of mechanisms and protective factors. J. Child. Psychol. Psychiatry.

[bib98] Rakesh D., McLaughlin K.A., Sheridan M., Humphreys K.L., Rosen M.L. (2024). Environmental contributions to cognitive development: The role of cognitive stimulation. Dev. Rev. DR.

[bib99] Rakesh D., Sadikova E., McLaughlin K. (2024). Beyond the income-achievement gap: The role of individual, family, and environmental factors in cognitive resilience among low-income youth. JCPP Adv..

[bib100] Rakesh D., Seguin C., Zalesky A., Cropley V., Whittle S. (2021). Associations Between Neighborhood Disadvantage, Resting-State Functional Connectivity, and Behavior in the Adolescent Brain Cognitive Development Study: The Moderating Role of Positive Family and School Environments. Biol. Psychiatry Cogn. Neurosci. Neuroimaging.

[bib101] Rakesh D., Tsomokos D.I., Vargas T., Pickett K.E., Patel V. (2025). Macroeconomic income inequality, brain structure and function, and mental health. Nat. Ment. Health.

[bib102] Rakesh D., Whittle S. (2021). Socioeconomic status and the developing brain—A systematic review of neuroimaging findings in youth. Neurosci. Biobehav. Rev..

[bib103] Rakesh D., Zalesky A., Whittle S. (2021). Similar but distinct – Effects of different socioeconomic indicators on resting state functional connectivity: Findings from the Adolescent Brain Cognitive Development (ABCD) Study®. Dev. Cogn. Neurosci..

[bib104] Rakesh D., Zalesky A., Whittle S. (2022). Assessment of Parent Income and Education, Neighborhood Disadvantage, and Child Brain Structure. JAMA Netw. Open..

[bib105] Rasmussen M.K., Mestre H., Nedergaard M. (2022). Fluid transport in the brain. Physiol. Rev..

[bib106] Reiss F., Meyrose A.-K., Otto C., Lampert T., Klasen F., Ravens-Sieberer U. (2019). Socioeconomic status, stressful life situations and mental health problems in children and adolescents: Results of the German BELLA cohort-study. PLOS One.

[bib107] Rey A.E., Guignard-Perret A., Imler-Weber F., Garcia-Larrea L., Mazza S. (2020). Improving sleep, cognitive functioning and academic performance with sleep education at school in children. Learn. Instr..

[bib108] Román-Caballero R., Vadillo M.A., Trainor L.J., Lupiáñez J. (2022). Please don’t stop the music: A meta-analysis of the cognitive and academic benefits of instrumental musical training in childhood and adolescence. Educ. Res. Rev..

[bib109] Romer A.L., Ren B., Pizzagalli D.A. (2023). Brain structure relations with psychopathology trajectories in the ABCD study. J. Am. Acad. Child. Adolesc. Psychiatry.

[bib110] Rosen M.L., Amso D., McLaughlin K.A. (2019). The role of the visual association cortex in scaffolding prefrontal cortex development: a novel mechanism linking socioeconomic status and executive function. Dev. Cogn. Neurosci..

[bib111] Rosen M.L., Hagen M.P., Lurie L.A., Miles Z.E., Sheridan M.A., Meltzoff A.N., McLaughlin K.A. (2020). Cognitive stimulation as a mechanism linking socioeconomic status with executive function: a longitudinal investigation. Child. Dev..

[bib112] Rosen M.L., Rakesh D., Romeo R.R. (2025). The role of socioeconomic status in shaping associations between sensory association cortex and prefrontal structure and implications for executive function. Dev. Cogn. Neurosci..

[bib113] Rosen M.L., Sheridan M.A., Sambrook K.A., Meltzoff A.N., McLaughlin K.A. (2018). Socioeconomic disparities in academic achievement: a multi-modal investigation of neural mechanisms in children and adolescents. NeuroImage.

[bib114] Saavedra J.M., Þorgeirsson S., Reykjavik University, School of Science and Engineering, Sports Science Department, Physical Activity, Physical Education, Health and Sport Research Centre (PAPESH), Reykjavik, Iceland, Kristjansdottir H., Reykjavik University, School of Science and Engineering, Sports Science Department, Physical Activity, Physical Education, Health and Sport Research Centre (PAPESH), Reykjavik, Iceland, Halldorsson K., Reykjavik University, School of Science and Engineering, Sports Science Department, Physical Activity, Physical Education, Health and Sport Research Centre (PAPESH), Reykjavik, Iceland, Guðmundsdottir M.L., Reykjavik University, School of Science and Engineering, Sports Science Department, Physical Activity, Physical Education, Health and Sport Research Centre (PAPESH), Reykjavik, Iceland, Einarsson I. Þ, Reykjavik University, School of Science and Engineering, Sports Science Department, Physical Activity, Physical Education, Health and Sport Research Centre (PAPESH), Reykjavik, Iceland (2018). Comparison of training volumes in different elite sportspersons according to sex, age, and sport practised. Monte J. Sports Sci. Med..

[bib115] Sadeh A., Gruber R., Raviv A. (2003). The effects of sleep restriction and extension on school-age children: what a difference an hour makes. Child. Dev..

[bib116] Sampson R.J., Morenoff J.D., Gannon-Rowley T. (2002). Assessing “Neighborhood Effects”: Social Processes and New Directions in Research. Annu. Rev. Sociol..

[bib117] Sanders A.F.P., Harms M.P., Kandala S., Marek S., Somerville L.H., Bookheimer S.Y., Dapretto M., Thomas K.M., Van Essen D.C., Yacoub E., Barch D.M. (2023). Age-related differences in resting-state functional connectivity from childhood to adolescence. Cereb. Cortex.

[bib118] Sauer J.D., Drummond A. (2020). Boundary Conditions for the Practical Importance of Small Effects in Long Runs: A Comment on Funder and Ozer (2019). Adv. Methods Pract. Psychol. Sci..

[bib119] Shibata M., Fuchino Y., Naoi N., Kohno S., Kawai M., Okanoya K., Myowa-Yamakoshi M. (2012). Broad cortical activation in response to tactile stimulation in newborns. NeuroReport.

[bib120] Snellman K., Silva J.M., Frederick C.B., Putnam R.D. (2015). The engagement gap: social mobility and extracurricular participation among american youth. Ann. Am. Acad. Political Social. Sci..

[bib121] Solé-Padullés C., Castro-Fornieles J., de la Serna E., Calvo R., Baeza I., Moya J., Lázaro L., Rosa M., Bargalló N., Sugranyes G. (2016). Intrinsic connectivity networks from childhood to late adolescence: Effects of age and sex. Developm. Cogn. Neurosci..

[bib122] Stevens M.C. (2016). The contributions of resting state and task-based functional connectivity studies to our understanding of adolescent brain network maturation. Neurosci. Biobehav. Rev. Adolesc. Brain.

[bib123] Sun L., Zhou H., Cichon J., Yang G. (2020). Experience and sleep-dependent synaptic plasticity: From structure to activity. Philos. Trans. R. Soc. B Biol. Sci..

[bib124] Tamnes C.K., Herting M.M., Goddings A.-L., Meuwese R., Blakemore S.-J., Dahl R.E., Güroğlu B., Raznahan A., Sowell E.R., Crone E.A., Mills K.L. (2017). Development of the cerebral cortex across adolescence: a multisample study of inter-related longitudinal changes in cortical volume, surface area, and thickness. J. Neurosci. Off. J. Soc. Neurosci..

[bib125] Trawick-Smith J. (2014).

[bib126] Tsomokos D.I., McLaughlin K.A., Whittle S., Dhamala E., Mehta M.A., Rakesh D. (2025). Socioeconomic disadvantage, pubertal and brain development, and internalizing problems in adolescence: a longitudinal investigation. Biol. Psychiatry: Cogn. Neurosci. Neuroimaging.

[bib127] White K.M., Ward L., Saelee R., Buxton O.M., Hale L., Chang A.-M., Johnson D.A. (2026). Measurement Bias in Adolescent Sleep Duration: Comparison of self-reported and actigraphy-assessed sleep duration among adolescents in the Future of Families and Child-Wellbeing Study. Sleep. Health.

[bib128] Whittle S., Vijayakumar N., Simmons J.G., Dennison M., Schwartz O., Pantelis C., Sheeber L., Byrne M.L., Allen N.B. (2017). Role of Positive Parenting in the Association Between Neighborhood Social Disadvantage and Brain Development Across Adolescence. JAMA Psychiatry.

[bib129] Yogman M., Garner A., Hutchinson J., Hirsh-Pasek K., Golinkoff R.M., Committee on psychosocial aspects of child and family health, & council on communications and media (2018). The Power of Play: A Pediatric Role in Enhancing Development in Young Children. Pediatrics.

[bib130] Zarazaga-Peláez J., Barrachina V., Gutiérrez-Logroño A., Villanueva-Guerrero O., Roso-Moliner A., Mainer-Pardos E. (2024). Impact of Extracurricular Physical Activity on Achievement of the Sustainable Development Goals and Academic Performance: Mediating Cognitive, Psychological, and Social Factors. Sustainability.

[bib131] Zheng J., Berg E., Byrne M.L., Rakesh D. (2025). Associations of Sleep, Screen Time, and Extracurricular Activities With Cognitive Development: A Longitudinal Study. J. Adolesc..

[bib132] Zhou L., Cai T., Ip K.I. (2025). Association Between Neighborhood Opportunity, Cognitive Function, and Brain Structure in Youths. Biol. Psychiatry Glob. Open. Sci..

