# Supplementary material for: Sleep and participation in extracurricular activities moderate associations between low SES and brain structure and functional connectivity
Source: Dev Cogn Neurosci. 2026 Sep 28;82:101827. doi: 10.1016/j.dcn.2026.101827 (PMC13658861; doi:10.1016/j.dcn.2026.101827)
Supplement: Supplementary file 1 [file mmc1.docx]

**Modifiable Lifestyle Factors in the Socioeconomic Status - Adolescent Brain Relationship**

Contents

[Supplementary Methods	4](#_Toc237769498)

[Household income bands for calculation of income-to-needs ratio	4](#_Toc237769499)

[Categorization of cortical regions into sensory, somatosensory/motor, and higher-order association systems	4](#_Toc237769500)

[Activities included in the physical and non-physical extracurricular activity calculation	5](#_Toc237769501)

[Model equation examples	5](#_Toc237769502)

[Figure S1. Histograms of SES variables: Area Deprivation Index (ADI), Income to needs ratio (INR) and parental education.	7](#_Toc237769503)

[Figure S2. Histograms of modifiable lifestyle variables: sleep duration, physical extracurricular activity, and non-physical extracurricular activity.	8](#_Toc237769504)

[Figure S3. Bivariate correlations for predictors and covariate (parental education).	9](#_Toc237769505)

[Figure S4. Joint distributions of lifestyle factors across neighborhood disadvantage.	9](#_Toc237769506)

[Figure S5. Joint distributions of lifestyle factors across income-to-needs ratio.	10](#_Toc237769507)

[Supplementary Results	11](#_Toc237769508)

[Table S1. Interaction effects between parental educational attainment and modifiable lifestyle factors (sleep duration, physical extracurricular activity, and non-physical extracurricular activity) in models of brain structure and function	11](#_Toc237769509)

[Table S2. Interaction effects between the latent household SES variable and modifiable lifestyle factors (sleep duration, physical extracurricular activity, and non-physical extracurricular activity) in models of brain structure and function	13](#_Toc237769510)

[Table S3. Interaction effects between socioeconomic status (neighborhood disadvantage and income-to-needs ratio) and unwinsorized physical extracurricular activity in models of brain structure and function	14](#_Toc237769511)

[Table S4. Comparison of sociodemographic measures and key measures of interest by youth with and without structural MRI data (unequal variances assumed).	17](#_Toc237769512)

[Table S5. Comparison of sociodemographic measures and key measures of interest by youth with and without fMRI data (unequal variances assumed).	18](#_Toc237769513)

[Table S6. Interaction effects between socioeconomic status (neighborhood disadvantage and income-to-needs ratio) and sleep duration in models of brain structure and function	20](#_Toc237769514)

[Table S7. Interaction effects between socioeconomic status (neighborhood disadvantage and income-to-needs ratio) and winsorized physical extracurricular activity in models of brain structure and function	22](#_Toc237769515)

[Table S8. Interaction effects between socioeconomic status (neighborhood disadvantage and income-to-needs ratio) and non-physical extracurricular activity in models of brain structure and function	24](#_Toc237769516)

[Figure S6. Moderating role of sleep duration (hours/night) in the associations between income-to-needs ratio (INR) and brain measures.	26](#_Toc237769517)

[Table S9. Simple slopes of significant interaction effects between modifiable factors and SES with SES factor as the moderator	28](#_Toc237769518)

[Figure S7. Association between sleep and brain measures at different levels of income-to-needs ratio (INR).	31](#_Toc237769519)

[Figure S8. Association between sleep and resting-state functional connectivity (rsFC) at different levels of area deprivation index (ADI).	33](#_Toc237769520)

[Figure S9. Association between physical extracurricular activity and brain structure at different levels of income-to-needs ratio (INR).	34](#_Toc237769521)

[Figure S10. Association between non-physical extracurricular activity and total volume (vol) of sensory cortices at different levels of income-to-needs ratio (INR).	35](#_Toc237769522)

[Table S10. Interaction effects between socioeconomic status (neighborhood disadvantage and income-to-needs ratio) and modifiable lifestyle factors in models of brain structure and function with site included as a random effect.	36](#_Toc237769523)

[Figure S11. Full range association between income-to-needs ratio (INR) and brain measures. At different levels of sleep.	38](#_Toc237769524)

[Figure S12. Full range association between area deprivation index (ADI) and resting-state functional connectivity (rsFC) at different levels of sleep	40](#_Toc237769525)

[Figure S13. Full range association between income-to-needs ratio (INR) and brain structure at different levels of physical extracurricular activity	41](#_Toc237769526)

[Figure S14. Full range association between income-to-needs ratio (INR) and total volume (vol) of sensory cortices at different levels of non-physical extracurricular activity.	42](#_Toc237769527)

[Table S11. Interaction effects between socioeconomic status (neighborhood disadvantage and income-to-needs ratio) and quadratic sleep duration in models of brain structure and function.	43](#_Toc237769528)

[Table S12. Distributions of Moderators across SES (neighborhood disadvantage and income-to-needs ratio) tertiles.	46](#_Toc237769529)

# Supplementary Methods

## Household income bands for calculation of income-to-needs ratio

Household SES was operationalized using the income-to-needs ratio (INR), calculated by dividing the median value of the total household income band by the 2017 federal poverty guideline for the respective household size (U.S. Department of Health & Human Services, 2017). The total household income bands used to calculate the median values were as follows:

1 = Less than $5,000 (Med = 5,000)

2 = $5,000 to $11,999 (Med = 8,500)

3 = $12,000 to $15,999 (Med = 13,999)

4 = $16,000 to $24,999 (Med = 20,500)

5 = $25,000 to $34,999 (Med = 30,000)

6 = $35,000 to $49,999 (Med = 42,500)

7 = $50,000 to $74,999 (Med = 62,500)

8 = $75,000 to $99,999 (Med = 87,500)

9 = $100,000 to $199,999 (Med = 150,000)

10 = $200,000 and greater (Med = 200,000)

## Categorization of cortical regions into sensory, somatosensory/motor, and higher-order association systems

For regional structural MRI analysis, cortical regions of the Desikan-Killiany atlas (Desikan et al., 2006) were categorized into sensory, somatosensory/motor, and higher-order association systems with the following assignments:

Sensory regions: inferior temporal, middle temporal, superior temporal transverse temporal, supra marginal, pericalcarine, fusiform, cuneus, lateral occipital, lingual

Somatosensory/motor regions: precentral, paracentral, postcentral gyrus

Higher-order association regions: caudal middle frontal, frontal pole, lateral orbitofrontal, medial orbitofrontal, pars opercularis, pars orbitalis, pars triangularis, rostral middle frontal, bankssts, entorhinal, inferior parietal, precuneus, isthmus cingulate, insula, parahippocampal, posterior cingulate, superior parietal

## Activities included in the physical and non-physical extracurricular activity calculation

Total weekly duration of physical extracurricular activity and total weekly duration of non-physical extracurricular activity were obtained by summing total weekly durations across all activities in each for the following categories:

Physical activities: Ballet/dance, baseball/softball, basketball, climbing, field hockey, football, gymnastics, ice hockey, horseback riding/polo, ice or inline skating, martial arts, lacrosse, rugby, skateboarding, skiing/snowboarding, soccer, surfing, swimming/water polo, tennis, track/running/cross-country, wrestling/mixed martial arts, volleyball, yoga/Tai Chi.

Non-physical activities: Musical instruments (singing/choir/guitar/piano/drums/violin/flute/band/rock band/orchestra), drawing/painting/graphic art/photography/pottery/sculpting, drama/theatre/acting/film, crafts like knitting/building model cars or airplanes, competitive games like chess/cards/darts.

## Model equation examples

Linear mixed effect models (*lmer* package in R version 2024.12.1) were used to evaluate whether the modifiable factors sleep, physical activity, and non-physical extracurricular activities moderate the association between SES (INR and area deprivation index [ADI]) and adolescent brain structure and resting-state functional connectivity (rsFC). Example model equations are provided for brain structure (total volume) and function (average between network rsFC) below.

Example brain structure model equations:

Total volume ~ INR * sleep + sex + age + parental education + scanner model + (1 | family ID)

Total volume ~ INR * physical activity + sex + age + parental education + scanner model + (1 | family ID)

Total volume ~ INR * non-physical activity + sex + age + parental education + scanner model + (1 | family ID)

Total volume ~ ADI * sleep + sex + age + parental education + scanner model + (1 | family ID)

Total volume ~ ADI * physical activity + sex + age + parental education + scanner model + (1 | family ID)

Total volume ~ ADI * non-physical activity + sex + age + parental education + scanner model + (1 | family ID)

Example rsFC model equations:

Average between-network rsFC ~ INR * sleep + sex + age + parental education + scanner model + mean framewise displacement + (1 | family ID)

Average between-network rsFC ~ INR * physical activity + sex + age + parental education + scanner model + mean framewise displacement + (1 | family ID)

Average between-network rsFC ~ INR * non-physical activity + sex + age + parental education + scanner model + mean framewise displacement + (1 | family ID)

Average between-network rsFC ~ ADI * sleep + sex + age + parental education + scanner model + mean framewise displacement + (1 | family ID)

Average between-network rsFC ~ ADI * physical activity + sex + age + parental education + scanner model + mean framewise displacement + (1 | family ID)

Average between-network rsFC ~ ADI * non-physical activity + sex + age + parental education + scanner model + mean framewise displacement + (1 | family ID)

## Figure S1. Histograms of SES variables: Area Deprivation Index (ADI), Income to needs ratio (INR) and parental education.

## Figure S2. Histograms of modifiable lifestyle variables: sleep duration, physical extracurricular activity, and non-physical extracurricular activity.

## Figure S3. Bivariate correlations for predictors and covariate (parental education).

## Figure S4. Distributions of lifestyle factors across neighborhood disadvantage. Violin and boxplots of sleep duration, physical activity, and non-physical activity across ADI tertiles. ADI = area deprivation index.

## Figure S5. Distributions of lifestyle factors across income-to-needs ratio. Violin and boxplots of sleep duration, physical activity, and non-physical activity across INR tertiles. INR = income-to-needs ratio.

# Supplementary Results

## Table S1. Interaction effects between parental educational attainment and modifiable lifestyle factors (sleep duration, physical extracurricular activity, and non-physical extracurricular activity) in models of brain structure and function

|  |  | Edu x Sleep duration | | | | | | Edu x Physical Extracurricular Activity (winsorized) | | | | | | Edu x Non-Physical Extracurricular Activity | | | | | |
| --- | --- | --- | --- | --- | --- | --- | --- | --- | --- | --- | --- | --- | --- | --- | --- | --- | --- | --- | --- |
| Outcome |  | b | SE | df | t | p | Adj.p | b | SE | df | t | p | Adj.p | b | SE | df | t | p | Adj.p |
| Total surface area | Total | -0.008 | 0.009 | 10,087 | -0.90 | 0.367 | 0.666 | -0.007 | 0.009 | 10,069 | -0.74 | 0.460 | 0.664 | -0.009 | 0.009 | 10,078 | -1.03 | 0.303 | 0.606 |
|  | Sensory | -0.007 | 0.009 | 10,079 | -0.77 | 0.439 | 0.666 | -0.009 | 0.009 | 10,056 | -0.96 | 0.336 | 0.576 | -0.009 | 0.009 | 10,071 | -1.05 | 0.295 | 0.606 |
|  | Somatosensory/ motor | -0.005 | 0.009 | 10,002 | -0.51 | 0.611 | 0.666 | 0.000 | 0.009 | 9,975 | -0.01 | 0.996 | 0.996 | -0.006 | 0.009 | 10,012 | -0.70 | 0.484 | 0.606 |
|  | Higher-order association | -0.006 | 0.010 | 9,718 | -0.59 | 0.558 | 0.666 | -0.006 | 0.010 | 9,701 | -0.68 | 0.498 | 0.664 | -0.007 | 0.009 | 9,727 | -0.77 | 0.443 | 0.606 |
| Total volume | Total | -0.008 | 0.009 | 10,115 | -0.92 | 0.357 | 0.666 | -0.012 | 0.009 | 10,101 | -1.27 | 0.206 | 0.576 | -0.012 | 0.009 | 10,103 | -1.33 | 0.184 | 0.606 |
|  | Sensory | -0.006 | 0.009 | 10,080 | -0.62 | 0.535 | 0.666 | -0.014 | 0.009 | 10,062 | -1.52 | 0.128 | 0.576 | -0.017 | 0.009 | 9,938 | -1.85 | 0.064 | 0.606 |
|  | Somatosensory/ motor | -0.005 | 0.010 | 10,006 | -0.52 | 0.600 | 0.666 | 0.000 | 0.010 | 9,983 | 0.01 | 0.988 | 0.996 | -0.010 | 0.010 | 10,017 | -1.10 | 0.272 | 0.606 |
|  | Higher-order association | -0.007 | 0.010 | 10,065 | -0.68 | 0.497 | 0.666 | -0.011 | 0.010 | 10,048 | -1.12 | 0.264 | 0.576 | -0.008 | 0.009 | 10,061 | -0.87 | 0.382 | 0.606 |
| Average cortical thickness | Total | -0.008 | 0.010 | 9,891 | -0.79 | 0.430 | 0.666 | -0.011 | 0.010 | 9,854 | -1.12 | 0.264 | 0.576 | -0.006 | 0.010 | 9,936 | -0.64 | 0.521 | 0.606 |
|  | Sensory | -0.012 | 0.010 | 9,923 | -1.15 | 0.249 | 0.666 | -0.010 | 0.010 | 9,890 | -1.00 | 0.320 | 0.576 | -0.001 | 0.010 | 9,958 | -0.09 | 0.925 | 0.925 |
|  | Somatosensory/ motor | -0.016 | 0.010 | 9,868 | -1.54 | 0.125 | 0.666 | -0.006 | 0.010 | 9,828 | -0.54 | 0.587 | 0.705 | -0.009 | 0.010 | 9,923 | -0.95 | 0.340 | 0.606 |
|  | Higher-order association | 0.000 | 0.010 | 9,874 | -0.01 | 0.995 | 0.995 | -0.018 | 0.010 | 9,833 | -1.70 | 0.088 | 0.576 | 0.006 | 0.010 | 9,926 | 0.59 | 0.556 | 0.606 |
| Average within-network rsFC | Total | -0.005 | 0.010 | 8,103 | -0.49 | 0.627 | 0.627 | 0.009 | 0.010 | 8,064 | 0.89 | 0.374 | 0.768 | 0.008 | 0.010 | 8,087 | 0.81 | 0.419 | 0.771 |
|  | Sensory | -0.005 | 0.011 | 8,058 | -0.52 | 0.606 | 0.627 | 0.010 | 0.011 | 8,041 | 0.91 | 0.364 | 0.768 | 0.003 | 0.011 | 8,106 | 0.29 | 0.771 | 0.771 |
|  | Somatosensory/ motor | 0.021 | 0.011 | 7,993 | 1.83 | 0.067 | 0.247 | 0.004 | 0.011 | 7,986 | 0.39 | 0.698 | 0.768 | 0.009 | 0.011 | 8,066 | 0.76 | 0.449 | 0.771 |
|  | Higher-order association | -0.013 | 0.010 | 8,142 | -1.32 | 0.186 | 0.428 | 0.007 | 0.010 | 8,097 | 0.67 | 0.505 | 0.768 | 0.006 | 0.010 | 8,103 | 0.64 | 0.521 | 0.771 |
| Average between-network rsFC | Total | 0.023 | 0.010 | 8,103 | 2.35 | 0.019 | 0.145 | 0.005 | 0.010 | 8,071 | 0.50 | 0.616 | 0.768 | 0.008 | 0.010 | 8,105 | 0.80 | 0.427 | 0.771 |
|  | Sensory | 0.009 | 0.011 | 8,090 | 0.79 | 0.430 | 0.591 | -0.008 | 0.012 | 8,075 | -0.69 | 0.489 | 0.768 | -0.016 | 0.011 | 8,123 | -1.43 | 0.153 | 0.771 |
|  | Sensory – somatosensory/ motor | 0.025 | 0.011 | 8,091 | 2.22 | 0.026 | 0.145 | 0.018 | 0.011 | 8,062 | 1.60 | 0.110 | 0.751 | 0.004 | 0.011 | 8,098 | 0.35 | 0.729 | 0.771 |
|  | Sensory – higher-order association | 0.014 | 0.011 | 8,083 | 1.30 | 0.195 | 0.428 | -0.009 | 0.011 | 8,055 | -0.82 | 0.414 | 0.768 | 0.005 | 0.011 | 8,094 | 0.42 | 0.671 | 0.771 |
|  | Somatosensory/ motor | 0.011 | 0.011 | 8,023 | 1.01 | 0.315 | 0.495 | 0.017 | 0.012 | 8,003 | 1.49 | 0.137 | 0.751 | 0.021 | 0.011 | 8,057 | 1.81 | 0.071 | 0.771 |
|  | Somatosensory/ motor – higher-order association | 0.005 | 0.010 | 7,990 | 0.52 | 0.601 | 0.627 | 0.004 | 0.011 | 7,973 | 0.41 | 0.678 | 0.768 | 0.013 | 0.011 | 8,042 | 1.20 | 0.230 | 0.771 |
|  | Higher-order association | 0.011 | 0.010 | 8,066 | 1.11 | 0.268 | 0.492 | 0.000 | 0.010 | 8,044 | 0.05 | 0.963 | 0.963 | -0.003 | 0.010 | 8,097 | -0.33 | 0.739 | 0.771 |

Note: The b values represent standardized b coefficients. Edu = parental educational attainment. SE = standard error. df = degrees of freedom. Adj. p = false-discovery rate corrected p value.

## Table S2. Interaction effects between the latent household SES variable and modifiable lifestyle factors (sleep duration, physical extracurricular activity, and non-physical extracurricular activity) in models of brain structure and function

|  |  | Household SES x Sleep duration | | | | | | Household x Physical Extracurricular Activity (winsorized) | | | | | | Household x Non-Physical Extracurricular Activity | | | | | |
| --- | --- | --- | --- | --- | --- | --- | --- | --- | --- | --- | --- | --- | --- | --- | --- | --- | --- | --- | --- |
| Outcome |  | b | SE | df | t | p | Adj.p | b | SE | df | t | p | Adj.p | b | SE | df | t | p | Adj.p |
| Total surface area | Total | 0.020 | 0.009 | 10,027 | 2.25 | 0.024 | 0.073 | 0.014 | 0.009 | 9,978 | 1.63 | 0.104 | 0.250 | 0.014 | 0.008 | 10,082 | 1.70 | 0.089 | 0.263 |
|  | Sensory | 0.020 | 0.009 | 10,016 | 2.29 | 0.022 | 0.073 | 0.018 | 0.009 | 9,966 | 2.08 | 0.038 | 0.227 | 0.016 | 0.008 | 10,074 | 1.93 | 0.053 | 0.213 |
|  | Somatosensory/ motor | 0.015 | 0.009 | 9,929 | 1.70 | 0.089 | 0.107 | 0.004 | 0.009 | 9,861 | 0.44 | 0.657 | 0.724 | 0.008 | 0.008 | 10,018 | 1.01 | 0.312 | 0.410 |
|  | Higher-order association | 0.017 | 0.009 | 9,652 | 1.83 | 0.067 | 0.089 | 0.012 | 0.009 | 9,613 | 1.37 | 0.169 | 0.290 | 0.011 | 0.008 | 9,734 | 1.32 | 0.188 | 0.322 |
| Total volume | Total | 0.021 | 0.009 | 10,063 | 2.38 | 0.017 | 0.073 | 0.016 | 0.009 | 10,022 | 1.80 | 0.072 | 0.243 | 0.017 | 0.008 | 10,106 | 2.07 | 0.039 | 0.213 |
|  | Sensory | 0.022 | 0.009 | 10,026 | 2.44 | 0.015 | 0.073 | 0.022 | 0.009 | 9,975 | 2.53 | 0.011 | 0.137 | 0.024 | 0.008 | 9,935 | 2.83 | 0.005 | 0.056 |
|  | Somatosensory/ motor | 0.018 | 0.010 | 9,933 | 1.88 | 0.060 | 0.089 | 0.004 | 0.009 | 9,871 | 0.41 | 0.683 | 0.724 | 0.013 | 0.009 | 10,023 | 1.51 | 0.131 | 0.263 |
|  | Higher-order association | 0.018 | 0.009 | 10,000 | 1.94 | 0.053 | 0.089 | 0.016 | 0.009 | 9,950 | 1.75 | 0.081 | 0.243 | 0.013 | 0.008 | 10,065 | 1.57 | 0.117 | 0.263 |
| Average cortical thickness | Total | 0.012 | 0.010 | 9,808 | 1.22 | 0.221 | 0.241 | 0.009 | 0.009 | 9,719 | 0.95 | 0.344 | 0.459 | 0.008 | 0.009 | 9,947 | 0.95 | 0.341 | 0.410 |
|  | Sensory | 0.019 | 0.010 | 9,841 | 1.90 | 0.057 | 0.089 | 0.010 | 0.009 | 9,761 | 1.07 | 0.286 | 0.429 | 0.005 | 0.009 | 9,967 | 0.57 | 0.571 | 0.623 |
|  | Somatosensory/ motor | 0.020 | 0.010 | 9,784 | 2.02 | 0.044 | 0.089 | 0.003 | 0.010 | 9,691 | 0.35 | 0.724 | 0.724 | 0.011 | 0.009 | 9,934 | 1.19 | 0.234 | 0.351 |
|  | Higher-order association | -0.003 | 0.010 | 9,791 | -0.25 | 0.802 | 0.802 | 0.015 | 0.010 | 9,697 | 1.53 | 0.127 | 0.254 | 0.000 | 0.009 | 9,938 | -0.05 | 0.957 | 0.957 |
| Average within-network rsFC | Total | 0.015 | 0.010 | 8,035 | 1.54 | 0.124 | 0.195 | -0.008 | 0.009 | 7,944 | -0.81 | 0.418 | 0.887 | -0.002 | 0.009 | 8,135 | -0.26 | 0.791 | 0.996 |
|  | Sensory | 0.011 | 0.010 | 7,995 | 1.10 | 0.272 | 0.374 | -0.008 | 0.010 | 7,911 | -0.83 | 0.405 | 0.887 | 0.000 | 0.010 | 8,159 | 0.00 | 0.996 | 0.996 |
|  | somatosensory/ motor | -0.022 | 0.011 | 7,924 | -2.06 | 0.039 | 0.107 | -0.007 | 0.011 | 7,841 | -0.70 | 0.486 | 0.887 | -0.009 | 0.010 | 8,126 | -0.84 | 0.402 | 0.737 |
|  | Higher-order association | 0.026 | 0.010 | 8,074 | 2.68 | 0.007 | 0.081 | -0.004 | 0.009 | 7,985 | -0.44 | 0.659 | 0.906 | 0.001 | 0.009 | 8,145 | 0.06 | 0.951 | 0.996 |
| Average between-network rsFC | Total | -0.022 | 0.009 | 8,039 | -2.33 | 0.020 | 0.107 | -0.001 | 0.009 | 7,952 | -0.09 | 0.927 | 0.991 | -0.016 | 0.009 | 8,152 | -1.80 | 0.072 | 0.263 |
|  | Sensory | -0.001 | 0.011 | 8,028 | -0.12 | 0.903 | 0.903 | 0.000 | 0.011 | 7,954 | -0.01 | 0.991 | 0.991 | 0.010 | 0.010 | 8,171 | 0.99 | 0.321 | 0.706 |
|  | Sensory – somatosensory/ motor | -0.019 | 0.011 | 8,026 | -1.77 | 0.076 | 0.168 | -0.023 | 0.011 | 7,941 | -2.21 | 0.027 | 0.300 | -0.005 | 0.010 | 8,146 | -0.49 | 0.627 | 0.985 |
|  | Sensory – higher-order association | -0.016 | 0.010 | 8,017 | -1.55 | 0.122 | 0.195 | 0.006 | 0.010 | 7,931 | 0.64 | 0.524 | 0.887 | -0.010 | 0.010 | 8,143 | -1.01 | 0.311 | 0.706 |
|  | Somatosensory/ motor | -0.023 | 0.011 | 7,952 | -2.15 | 0.032 | 0.107 | -0.017 | 0.011 | 7,866 | -1.58 | 0.113 | 0.622 | -0.021 | 0.010 | 8,111 | -2.04 | 0.042 | 0.230 |
|  | Somatosensory/ motor – higher-order association | -0.009 | 0.010 | 7,918 | -0.84 | 0.399 | 0.487 | 0.006 | 0.010 | 7,827 | 0.58 | 0.564 | 0.887 | -0.020 | 0.010 | 8,101 | -2.05 | 0.041 | 0.230 |
|  | Higher-order association | -0.007 | 0.010 | 8,001 | -0.67 | 0.505 | 0.555 | 0.003 | 0.010 | 7,916 | 0.30 | 0.766 | 0.936 | 0.000 | 0.009 | 8,148 | -0.02 | 0.988 | 0.996 |

Note: The b values represent standardized b coefficients. SE = standard error. df = degrees of freedom. Adj. p = false-discovery rate corrected p value.

## Table S3. Interaction effects between socioeconomic status (neighborhood disadvantage and income-to-needs ratio) and unwinsorized physical extracurricular activity in models of brain structure and function

|  | | ADI x Physical Extracurricular Activity (unwinsorized) | | | | | | INR x Physical Extracurricular Activity (unwinsorized) | | | | | |
| --- | --- | --- | --- | --- | --- | --- | --- | --- | --- | --- | --- | --- | --- |
| Outcome |  | b | SE | df | t | p | Adj.p | b | SE | df | t | p | Adj.p |
| Total surface area | Total | -0.007 | 0.008 | 10,211 | -0.95 | 0.343 | 0.584 | -0.019 | 0.008 | 9,847 | -2.27 | 0.023 | 0.069 |
|  | Sensory | -0.005 | 0.008 | 10,213 | -0.62 | 0.533 | 0.584 | -0.021 | 0.008 | 9,833 | -2.56 | 0.010 | 0.063 |
|  | Somatosensory/motor | -0.016 | 0.008 | 10,064 | -2.04 | 0.041 | 0.245 | -0.007 | 0.008 | 9,707 | -0.82 | 0.413 | 0.551 |
|  | Higher-order association | -0.005 | 0.008 | 9,846 | -0.67 | 0.501 | 0.584 | -0.019 | 0.009 | 9,483 | -2.15 | 0.031 | 0.070 |
| Total volume | Total | -0.011 | 0.008 | 10,271 | -1.48 | 0.140 | 0.559 | -0.018 | 0.008 | 9,902 | -2.11 | 0.035 | 0.070 |
|  | Sensory | -0.005 | 0.008 | 10,134 | -0.62 | 0.535 | 0.584 | -0.026 | 0.009 | 9,807 | -3.02 | 0.003 | 0.031* |
|  | Somatosensory/motor | -0.020 | 0.008 | 10,061 | -2.38 | 0.017 | 0.206 | -0.006 | 0.009 | 9,719 | -0.69 | 0.489 | 0.587 |
|  | Higher-order association | -0.009 | 0.008 | 10,165 | -1.10 | 0.273 | 0.584 | -0.020 | 0.009 | 9,811 | -2.28 | 0.023 | 0.069 |
| Average cortical thickness | Total | -0.007 | 0.008 | 9,875 | -0.87 | 0.386 | 0.584 | -0.005 | 0.009 | 9,542 | -0.51 | 0.613 | 0.669 |
|  | Sensory | -0.006 | 0.008 | 9,922 | -0.76 | 0.448 | 0.584 | -0.009 | 0.009 | 9,590 | -0.95 | 0.344 | 0.551 |
|  | Somatosensory/motor | -0.011 | 0.009 | 9,823 | -1.27 | 0.205 | 0.584 | 0.000 | 0.009 | 9,511 | 0.01 | 0.993 | 0.993 |
|  | Higher-order association | -0.003 | 0.009 | 9,872 | -0.35 | 0.728 | 0.728 | -0.008 | 0.009 | 9,519 | -0.89 | 0.373 | 0.551 |
| Average within-network rsFC | Total | -0.001 | 0.008 | 8,202 | -0.11 | 0.914 | 0.921 | 0.001 | 0.009 | 7,822 | 0.15 | 0.880 | 0.975 |
|  | Sensory | 0.004 | 0.009 | 8,166 | 0.44 | 0.657 | 0.921 | 0.004 | 0.010 | 7,789 | 0.37 | 0.711 | 0.975 |
|  | Somatosensory/motor | -0.009 | 0.010 | 8,111 | -0.96 | 0.338 | 0.921 | 0.010 | 0.010 | 7,710 | 0.97 | 0.334 | 0.936 |
|  | Higher-order association | 0.002 | 0.009 | 8,241 | 0.20 | 0.840 | 0.921 | -0.004 | 0.009 | 7,867 | -0.41 | 0.682 | 0.975 |
| Average between-network rsFC | Total | -0.001 | 0.008 | 8,226 | -0.10 | 0.921 | 0.921 | 0.000 | 0.009 | 7,836 | -0.03 | 0.974 | 0.975 |
|  | Sensory | -0.004 | 0.010 | 8,234 | -0.37 | 0.709 | 0.921 | 0.006 | 0.010 | 7,840 | 0.55 | 0.586 | 0.975 |
|  | Sensory – somatosensory/motor | -0.009 | 0.010 | 8,203 | -0.91 | 0.362 | 0.921 | 0.021 | 0.010 | 7,821 | 2.06 | 0.040 | 0.437 |
|  | Sensory – higher-order association | 0.005 | 0.009 | 8,180 | 0.52 | 0.605 | 0.921 | -0.002 | 0.010 | 7,810 | -0.23 | 0.818 | 0.975 |
|  | Somatosensory/motor | -0.007 | 0.010 | 8,115 | -0.68 | 0.496 | 0.921 | 0.010 | 0.010 | 7,735 | 0.95 | 0.340 | 0.936 |
|  | Somatosensory/motor – higher-order association | 0.010 | 0.009 | 8,104 | 1.07 | 0.285 | 0.921 | -0.013 | 0.010 | 7,690 | -1.37 | 0.171 | 0.936 |
|  | Higher-order association | -0.007 | 0.009 | 8,194 | -0.81 | 0.418 | 0.921 | 0.000 | 0.009 | 7,796 | 0.03 | 0.975 | 0.975 |

Note. Using unwinsorized data, the interaction between income-to-needs ratio (INR) and physical extracurricular activity was no longer significant for the surface area of sensory regions (observed in winsorized data; Table 2), but significant interaction effects persisted for sensory volume. As in winsorized data, no interactions between area deprivation index (ADI) and physical activity were significant for any brain feature. The b values represent standardized b coefficients. SE = standard error. df = degrees of freedom. Adj. p = false-discovery rate corrected p value. * = p < .05, ** = p < .01.

## Table S4. Comparison of sociodemographic measures and key measures of interest by youth with and without structural MRI data (unequal variances assumed).

| Characteristic | Included | Excluded | P-value |
| --- | --- | --- | --- |
| No. of participants (Female %) | 10171 (48.4 %) | 480 (41.2 %) | 0.002 |
| Age (years) | 10 (0.6) [8.3, 11.3] | 9.8 (0.6) [9, 11] | <0.001 |
| Income-to-reeds ratio | 3.7 (2.5) [0.1, 12.4] | 3.6 (2.5) [0.1, 12.3] | 0.174 |
| ADI | 38.9 (26.3) [1, 100] | 44.6 (29.1) [1, 100] | <0.001 |
| Average parental education (years) | 15.3 (2.5) [3, 22] | 15.2 (2.6) [4, 22] | 0.491 |
| Physical extracurricular activity (hours per week) | 6.9 (6.9) [0, 76.8] | 6.4 (6.9) [0, 43.5] | 0.08 |
| Non-physical extracurricular activity (hours per week) | 1.8 (3.2) [0, 36] | 1.7 (3.1) [0, 21] | 0.592 |
| Sleep duration (hours) | 9 (1.1) [5, 10] | 9 (1.1) [5, 10] | 0.251 |

Note: P-values based on independent-samples t-tests assuming unequal variances, or chi-squared tests for sex, between those with or without passing structural MRI (sMRI) data, after exclusion for those missing income-to-needs ratio and covariates (n=1,217).

## Table S5. Comparison of sociodemographic measures and key measures of interest by youth with and without fMRI data (unequal variances assumed).

| Characteristic | Included | Excluded | P-value |
| --- | --- | --- | --- |
| No. of participants (Female %) | 8499 (49.9 %) | 2152 (40.9 %) | <0.001 |
| Age (years) | 10 (0.6) [9, 11.3] | 9.9 (0.6) [8.3, 11.1] | <0.001 |
| Income-to-reeds ratio | 3.8 (2.4) [0.1, 12.4] | 3.5 (2.5) [0.1, 12.3] | <0.001 |
| ADI | 38.5 (25.9) [1, 100] | 42 (28.6) [1, 100] | <0.001 |
| Average parental education (years) | 15.3 (2.5) [3, 22] | 15 (2.6) [4, 22] | <0.001 |
| Physical extracurricular activity (hours per week) | 6.9 (6.8) [0, 76.8] | 6.8 (7.4) [0, 66] | 0.447 |
| Non-physical extracurricular activity (hours per week) | 1.8 (3.2) [0, 36] | 1.7 (3.3) [0, 31.8] | 0.246 |
| Sleep duration (hours) | 9.1 (1.1) [5, 10] | 9 (1.1) [5, 10] | <0.001 |

Note: P-values based on independent-samples t-tests assuming unequal variances, or chi-squared tests for sex, between those with or without passing functional MRI (fMRI) data, after exclusion for those missing income-to-needs ratio and covariates (n=1,217).

## Table S6. Interaction effects between socioeconomic status (neighborhood disadvantage and income-to-needs ratio) and sleep duration in models of brain structure and function

|  |  | ADI x Sleep Duration | | | | | | INR x Sleep Duration | | | | | |
| --- | --- | --- | --- | --- | --- | --- | --- | --- | --- | --- | --- | --- | --- |
| Outcome |  | b | SE | df | t | p | Adj.p | b | SE | df | t | p | Adj.p |
| Total surface area | Total | -0.001 | 0.008 | 10,189 | -0.17 | 0.868 | 0.939 | -0.029 | 0.009 | 9,937 | -3.21 | 0.001 | 0.004** |
|  | Sensory | 0.001 | 0.008 | 10,191 | 0.08 | 0.939 | 0.939 | -0.030 | 0.009 | 9,925 | -3.36 | 0.001 | 0.003** |
|  | Somatosensory/motor | 0.001 | 0.008 | 10,077 | 0.16 | 0.872 | 0.939 | -0.024 | 0.009 | 9,831 | -2.64 | 0.008 | 0.012* |
|  | Higher-order association | -0.001 | 0.008 | 9,802 | -0.12 | 0.906 | 0.939 | -0.026 | 0.009 | 9,550 | -2.80 | 0.005 | 0.009** |
| Total volume | Total | 0.003 | 0.008 | 10,238 | 0.33 | 0.741 | 0.939 | -0.031 | 0.009 | 9,980 | -3.43 | 0.001 | 0.003** |
|  | Sensory | 0.009 | 0.008 | 10,191 | 1.07 | 0.286 | 0.859 | -0.035 | 0.009 | 9,940 | -3.80 | 0.000 | 0.002** |
|  | Somatosensory/motor | 0.004 | 0.009 | 10,071 | 0.45 | 0.655 | 0.939 | -0.029 | 0.010 | 9,836 | -2.96 | 0.003 | 0.007** |
|  | Higher-order association | -0.001 | 0.008 | 10,149 | -0.08 | 0.939 | 0.939 | -0.027 | 0.009 | 9,907 | -2.89 | 0.004 | 0.008** |
| Average cortical thickness | Total | 0.013 | 0.009 | 9,940 | 1.46 | 0.144 | 0.759 | -0.014 | 0.010 | 9,707 | -1.43 | 0.154 | 0.168 |
|  | Sensory | 0.021 | 0.009 | 9,969 | 2.33 | 0.020 | 0.238 | -0.023 | 0.010 | 9,741 | -2.31 | 0.021 | 0.028* |
|  | Somatosensory/motor | 0.012 | 0.009 | 9,913 | 1.31 | 0.190 | 0.759 | -0.022 | 0.010 | 9,686 | -2.17 | 0.030 | 0.036* |
|  | Higher-order association | 0.004 | 0.009 | 9,945 | 0.44 | 0.657 | 0.939 | 0.005 | 0.010 | 9,693 | 0.46 | 0.647 | 0.647 |
| Average within-network rsFC | Total | 0.000 | 0.009 | 8,234 | 0.05 | 0.962 | 0.962 | -0.023 | 0.010 | 7,949 | -2.44 | 0.015 | 0.054 |
|  | Sensory | 0.008 | 0.009 | 8,203 | 0.83 | 0.409 | 0.500 | -0.016 | 0.010 | 7,924 | -1.54 | 0.124 | 0.208 |
|  | Somatosensory/motor | -0.023 | 0.010 | 8,139 | -2.35 | 0.019 | 0.069 | 0.021 | 0.011 | 7,850 | 1.91 | 0.057 | 0.133 |
|  | Higher-order association | 0.007 | 0.009 | 8,270 | 0.84 | 0.403 | 0.500 | -0.036 | 0.010 | 7,986 | -3.72 | 0.000 | 0.002** |
| Average between-network rsFC | Total | -0.027 | 0.008 | 8,255 | -3.22 | 0.001 | 0.014 | 0.017 | 0.009 | 7,961 | 1.88 | 0.061 | 0.133 |
|  | Sensory | -0.007 | 0.010 | 8,256 | -0.73 | 0.464 | 0.510 | -0.007 | 0.011 | 7,956 | -0.66 | 0.506 | 0.557 |
|  | Sensory – somatosensory/motor | -0.022 | 0.010 | 8,228 | -2.22 | 0.026 | 0.072 | 0.009 | 0.011 | 7,947 | 0.80 | 0.424 | 0.518 |
|  | Sensory – higher-order association | -0.009 | 0.009 | 8,211 | -1.00 | 0.318 | 0.500 | 0.016 | 0.010 | 7,939 | 1.50 | 0.133 | 0.208 |
|  | Somatosensory/motor | -0.026 | 0.010 | 8,135 | -2.63 | 0.009 | 0.047 | 0.030 | 0.011 | 7,869 | 2.74 | 0.006 | 0.034* |
|  | Somatosensory/motor – higher-order association | -0.008 | 0.009 | 8,132 | -0.87 | 0.384 | 0.500 | 0.011 | 0.010 | 7,834 | 1.11 | 0.267 | 0.368 |
|  | Higher-order association | -0.018 | 0.009 | 8,223 | -1.98 | 0.047 | 0.104 | 0.002 | 0.010 | 7,927 | 0.16 | 0.870 | 0.870 |

Note. The b values represent standardized b coefficients. ADI = area deprivation index. INR = income-to-needs ratio. SE = standard error. df = degrees of freedom. Adj. p = false-discovery rate corrected p value. * = p < .05, ** = p < .01.

## Table S7. Interaction effects between socioeconomic status (neighborhood disadvantage and income-to-needs ratio) and winsorized physical extracurricular activity in models of brain structure and function

| Outcome |  | ADI x Physical Extracurricular Activity (winsorized) | | | | | | INR x Physical Extracurricular Activity (winsorized) | | | | | |
| --- | --- | --- | --- | --- | --- | --- | --- | --- | --- | --- | --- | --- | --- |
|  |  | b | SE | df | t | p | Adj.p | b | SE | df | t | p | Adj.p |
| Total surface area | Total | -0.009 | 0.008 | 10,237 | -1.06 | 0.289 | 0.650 | -0.020 | 0.009 | 9,866 | -2.31 | 0.021 | 0.080 |
|  | Sensory | -0.005 | 0.008 | 10,238 | -0.61 | 0.544 | 0.652 | -0.024 | 0.009 | 9,852 | -2.77 | 0.006 | 0.034* |
|  | Somatosensory/motor | -0.016 | 0.008 | 10,106 | -1.89 | 0.058 | 0.349 | -0.009 | 0.009 | 9,733 | -1.02 | 0.306 | 0.372 |
|  | Higher-order association | -0.008 | 0.009 | 9,860 | -0.89 | 0.372 | 0.650 | -0.018 | 0.009 | 9,500 | -2.04 | 0.041 | 0.082 |
| Total volume | Total | -0.013 | 0.008 | 10,290 | -1.62 | 0.106 | 0.423 | -0.018 | 0.009 | 9,919 | -2.10 | 0.036 | 0.082 |
|  | Sensory | -0.006 | 0.008 | 10,220 | -0.68 | 0.498 | 0.652 | -0.027 | 0.009 | 9,870 | -3.09 | 0.002 | 0.024* |
|  | Somatosensory/motor | -0.019 | 0.009 | 10,103 | -2.09 | 0.037 | 0.349 | -0.009 | 0.009 | 9,745 | -1.02 | 0.310 | 0.372 |
|  | Higher-order association | -0.010 | 0.009 | 10,195 | -1.22 | 0.224 | 0.650 | -0.020 | 0.009 | 9,832 | -2.22 | 0.027 | 0.080 |
| Average cortical thickness | Total | -0.006 | 0.009 | 9,940 | -0.67 | 0.504 | 0.652 | -0.006 | 0.009 | 9,576 | -0.64 | 0.521 | 0.569 |
|  | Sensory | -0.005 | 0.009 | 9,982 | -0.53 | 0.598 | 0.652 | -0.010 | 0.009 | 9,622 | -1.06 | 0.290 | 0.372 |
|  | Somatosensory/motor | -0.008 | 0.009 | 9,895 | -0.88 | 0.379 | 0.650 | -0.003 | 0.009 | 9,548 | -0.32 | 0.750 | 0.750 |
|  | Higher-order association | 0.000 | 0.009 | 9,938 | 0.02 | 0.982 | 0.982 | -0.010 | 0.010 | 9,555 | -1.06 | 0.290 | 0.372 |
| Average within-network rsFC | Total | -0.001 | 0.009 | 8,227 | -0.12 | 0.907 | 0.973 | 0.002 | 0.009 | 7,838 | 0.17 | 0.865 | 0.869 |
|  | Sensory | 0.004 | 0.009 | 8,169 | 0.38 | 0.704 | 0.973 | 0.004 | 0.010 | 7,797 | 0.40 | 0.689 | 0.869 |
|  | Somatosensory/motor | -0.009 | 0.010 | 8,113 | -0.85 | 0.394 | 0.973 | 0.010 | 0.010 | 7,717 | 0.98 | 0.328 | 0.869 |
|  | Higher-order association | 0.002 | 0.009 | 8,272 | 0.17 | 0.866 | 0.973 | -0.004 | 0.009 | 7,886 | -0.41 | 0.681 | 0.869 |
| Average between-network rsFC | Total | 0.000 | 0.009 | 8,244 | -0.03 | 0.973 | 0.973 | -0.001 | 0.009 | 7,849 | -0.17 | 0.869 | 0.869 |
|  | Sensory | -0.003 | 0.010 | 8,245 | -0.27 | 0.785 | 0.973 | 0.005 | 0.011 | 7,850 | 0.48 | 0.631 | 0.869 |
|  | Sensory – somatosensory/motor | -0.007 | 0.010 | 8,218 | -0.72 | 0.472 | 0.973 | 0.021 | 0.010 | 7,835 | 2.02 | 0.043 | 0.476 |
|  | Sensory – higher-order association | 0.005 | 0.010 | 8,193 | 0.52 | 0.604 | 0.973 | -0.003 | 0.010 | 7,823 | -0.29 | 0.773 | 0.869 |
|  | Somatosensory/motor | -0.007 | 0.010 | 8,132 | -0.69 | 0.490 | 0.973 | 0.012 | 0.011 | 7,747 | 1.12 | 0.265 | 0.869 |
|  | Somatosensory/motor – higher-order association | 0.008 | 0.009 | 8,108 | 0.81 | 0.421 | 0.973 | -0.011 | 0.010 | 7,699 | -1.14 | 0.255 | 0.869 |
|  | Higher-order association | -0.005 | 0.009 | 8,206 | -0.58 | 0.564 | 0.973 | -0.003 | 0.009 | 7,806 | -0.35 | 0.729 | 0.869 |

Note. The b values represent standardized b coefficients. ADI = area deprivation index. INR = income-to-needs ratio. SE = standard error. df = degrees of freedom. Adj. p = false-discovery rate corrected p value. * = p < .05, ** = p < .01.

## Table S8. Interaction effects between socioeconomic status (neighborhood disadvantage and income-to-needs ratio) and non-physical extracurricular activity in models of brain structure and function

|  |  | ADI x Non-Physical Extracurricular Activity | | | | | | INR x Non-Physical Extracurricular Activity | | | | | |
| --- | --- | --- | --- | --- | --- | --- | --- | --- | --- | --- | --- | --- | --- |
| Outcome |  | b | SE | df | t | p | Adj.p | b | SE | df | t | p | Adj.p |
| Total surface area | Total | -0.001 | 0.008 | 10,446 | -0.12 | 0.905 | 0.984 | -0.016 | 0.008 | 10,121 | -2.09 | 0.036 | 0.104 |
|  | Sensory | -0.002 | 0.008 | 10,441 | -0.22 | 0.826 | 0.984 | -0.019 | 0.008 | 10,115 | -2.43 | 0.015 | 0.060 |
|  | Somatosensory/motor | -0.001 | 0.008 | 10,449 | -0.17 | 0.862 | 0.984 | -0.010 | 0.008 | 10,068 | -1.21 | 0.226 | 0.301 |
|  | Higher-order association | 0.000 | 0.008 | 10,080 | -0.02 | 0.984 | 0.984 | -0.014 | 0.008 | 9,771 | -1.70 | 0.088 | 0.151 |
| Total volume | Total | 0.004 | 0.008 | 10,428 | 0.45 | 0.653 | 0.984 | -0.019 | 0.008 | 10,139 | -2.45 | 0.014 | 0.060 |
|  | Sensory | 0.005 | 0.008 | 10,411 | 0.59 | 0.558 | 0.984 | -0.026 | 0.008 | 10,047 | -3.26 | 0.001 | 0.014* |
|  | Somatosensory/motor | 0.001 | 0.009 | 10,448 | 0.08 | 0.938 | 0.984 | -0.015 | 0.009 | 10,072 | -1.73 | 0.084 | 0.151 |
|  | Higher-order association | -0.001 | 0.008 | 10,448 | -0.14 | 0.887 | 0.984 | -0.017 | 0.008 | 10,108 | -2.02 | 0.043 | 0.104 |
| Average cortical thickness | Total | 0.002 | 0.009 | 10,419 | 0.22 | 0.824 | 0.984 | -0.009 | 0.009 | 10,001 | -1.09 | 0.274 | 0.328 |
|  | Sensory | 0.003 | 0.009 | 10,429 | 0.32 | 0.752 | 0.984 | -0.008 | 0.009 | 10,020 | -0.96 | 0.337 | 0.368 |
|  | Somatosensory/motor | -0.003 | 0.009 | 10,404 | -0.31 | 0.754 | 0.984 | -0.011 | 0.009 | 9,987 | -1.29 | 0.197 | 0.295 |
|  | Higher-order association | -0.004 | 0.009 | 10,416 | -0.40 | 0.687 | 0.984 | -0.004 | 0.009 | 9,989 | -0.46 | 0.644 | 0.644 |
| Average within-network rsFC | Total | 0.015 | 0.009 | 8,520 | 1.69 | 0.091 | 0.239 | -0.004 | 0.009 | 8,196 | -0.43 | 0.667 | 0.724 |
|  | Sensory | 0.010 | 0.009 | 8,545 | 1.07 | 0.287 | 0.394 | -0.003 | 0.009 | 8,213 | -0.35 | 0.724 | 0.724 |
|  | Somatosensory/motor | -0.002 | 0.010 | 8,527 | -0.16 | 0.871 | 0.871 | 0.008 | 0.010 | 8,184 | 0.81 | 0.416 | 0.724 |
|  | Higher-order association | 0.018 | 0.009 | 8,527 | 2.04 | 0.042 | 0.162 | -0.008 | 0.009 | 8,208 | -0.90 | 0.369 | 0.724 |
| Average between-network rsFC | Total | -0.017 | 0.008 | 8,538 | -2.01 | 0.044 | 0.162 | 0.021 | 0.009 | 8,209 | 2.45 | 0.014 | 0.080 |
|  | Sensory | 0.008 | 0.010 | 8,556 | 0.80 | 0.424 | 0.518 | -0.005 | 0.010 | 8,224 | -0.47 | 0.638 | 0.724 |
|  | Sensory –Somatosensory/motor | 0.016 | 0.010 | 8,536 | 1.60 | 0.109 | 0.239 | 0.004 | 0.010 | 8,204 | 0.42 | 0.678 | 0.724 |
|  | Sensory – higher-order association | -0.011 | 0.009 | 8,533 | -1.20 | 0.232 | 0.364 | 0.013 | 0.009 | 8,201 | 1.38 | 0.168 | 0.461 |
|  | Somatosensory/motor | -0.007 | 0.010 | 8,505 | -0.67 | 0.506 | 0.556 | 0.018 | 0.010 | 8,174 | 1.79 | 0.073 | 0.269 |
|  | Somatosensory/motor – higher-order association | -0.022 | 0.009 | 8,521 | -2.32 | 0.021 | 0.162 | 0.024 | 0.009 | 8,163 | 2.54 | 0.011 | 0.080 |
|  | Higher-order association | -0.011 | 0.009 | 8,538 | -1.20 | 0.231 | 0.364 | 0.004 | 0.009 | 8,204 | 0.41 | 0.683 | 0.724 |

Note. The b values represent standardized b coefficients. ADI = area deprivation index. INR = income-to-needs ratio. SE = standard error. df = degrees of freedom. Adj. p = false-discovery rate corrected p value. * = p < .05, ** = p < .01.

## Figure S6. Moderating role of sleep duration (hours/night) in the associations between income-to-needs ratio (INR) and brain measures.

Panels A to D show associations between INR and brain structure at different sleep levels for: (A) total volume (vol), (B) total surface area (SA), (C) total SA of sensory cortices , (D) total SA of somatosensory/motor cortices, (E) total SA of higher-order association cortices, (F) total vol of sensory cortices, (G) total vol of somatosensory/motor cortices, (H) total vol higher-order association cortices,(I) average cortical thickness (CT) of sensory cortices, (J) average CT of somatosensory/motor cortices. Panels K to L show associations between INR and resting-state functional connectivity (rsFC) at different levels of sleep for: (K) within-higher-order association network rsFC, and (L) between somatosensory/motor network rsFC. For structural outcomes (Panels A-J), the y-axis shows standardized values; for rsFC measures (Panels K-L), the y-axis shows values in their original units. Slopes represent predicted values at mean ± 1 SD of sleep, and shaded areas indicate 95% confidence intervals.

## Table S9. Simple slopes of significant interaction effects between modifiable factors and SES with SES factor as the moderator

| Sleep x ADI  Between rsFC | Moderator value | b value | p value |
| --- | --- | --- | --- |
|  | -1.0042 | 0.0286 | 0.036* |
|  | -0.0225 | 0.0023 | 0.808 |
|  | 0.9591 | -0.0241 | 0.029* |

| Sleep x ADI  between rsFC (sensorimotor) | Moderator value | b value | p value |
| --- | --- | --- | --- |
|  | -1.0042 | -0.0909 | 0.000*** |
|  | -0.0225 | -0.1168 | 0.000*** |
|  | 0.9591 | -0.1427 | 0.000*** |

| Sleep x INR  Total SA | Moderator value | b value | p value |
| --- | --- | --- | --- |
|  | -0.9952 | 0.0834 | 0.000*** |
|  | 0.0035 | 0.0548 | 0.000*** |
|  | 1.0022 | 0.0262 | 0.066 |

| Sleep x INR  SA (sensory) | Moderator value | b value | p value |
| --- | --- | --- | --- |
|  | -0.9948 | 0.0784 | 0.000*** |
|  | 0.0038 | 0.0483 | 0.000*** |
|  | 1.0025 | 0.0182 | 0.206 |

| Sleep x INR  SA (sensorimotor) | Moderator value | b value | p value |
| --- | --- | --- | --- |
|  | -0.9952 | 0.0722 | 0.000*** |
|  | 0.0035 | 0.0483 | 0.000*** |
|  | 1.0022 | 0.0243 | 0.095 |

| Sleep x INR  SA (higher-order association) | Moderator value | b value | p value |
| --- | --- | --- | --- |
|  | -0.9953 | 0.0924 | 0.000*** |
|  | 0.0034 | 0.0663 | 0.000*** |
|  | 1.0020 | 0.0402 | 0.007** |

| Sleep x INR  Total Vol | Moderator value | b value | p value |
| --- | --- | --- | --- |
|  | -0.9952 | 0.0957 | 0.000*** |
|  | 0.0035 | 0.0651 | 0.000*** |
|  | 1.0022 | 0.0344 | 0.017* |

| Sleep x INR  Vol (sensory) | Moderator value | b value | p value |
| --- | --- | --- | --- |
|  | -0.9946 | 0.1034 | 0.000*** |
|  | 0.0040 | 0.0688 | 0.000*** |
|  | 1.0026 | 0.0342 | 0.019* |

| Sleep x INR  Vol (sensorimotor) | Moderator value | b value | p value |
| --- | --- | --- | --- |
|  | -0.9952 | 0.0950 | 0.000*** |
|  | 0.0035 | 0.0662 | 0.000*** |
|  | 1.0022 | 0.0373 | 0.017* |

| Sleep x INR  Vol (higher-order association) | Moderator value | b value | p value |
| --- | --- | --- | --- |
|  | -0.9949 | 0.1049 | 0.000*** |
|  | 0.0038 | 0.0779 | 0.000*** |
|  | 1.0024 | 0.0509 | 0.001** |

| Sleep x INR  CT (sensory) | Moderator value | b value | p value |
| --- | --- | --- | --- |
|  | -0.9952 | 0.0860 | 0.000*** |
|  | 0.0035 | 0.0633 | 0.000*** |
|  | 1.0022 | 0.0407 | 0.010** |

| Sleep x INR  CT (sensorimotor) | Moderator value | b value | p value |
| --- | --- | --- | --- |
|  | -0.9952 | 0.0632 | 0.000*** |
|  | 0.0035 | 0.0417 | 0.000*** |
|  | 1.0022 | 0.0202 | 0.205 |

| Sleep x INR  Within rsFC (higher-order association) | Moderator value | b value | p value |
| --- | --- | --- | --- |
|  | -0.9661 | 0.0879 | 0.000*** |
|  | 0.0274 | 0.0522 | 0.000*** |
|  | 1.0209 | 0.0165 | 0.289 |

| Sleep x INR  Between rsFC (sensorimotor) | Moderator value | b value | p value |
| --- | --- | --- | --- |
|  | -0.9658 | -0.1486 | 0.000*** |
|  | 0.0277 | -0.1188 | 0.000*** |
|  | 1.0212 | -0.0891 | 0.000*** |

| PA x INR  SA (sensory) | Moderator value | b value | p value |
| --- | --- | --- | --- |
|  | -0.9948 | 0.0352 | 0.006** |
|  | 0.0038 | 0.0107 | 0.239 |
|  | 1.0024 | -0.0138 | 0.264 |

| PA x INR  Vol (sensory) | Moderator value | b value | p value |
| --- | --- | --- | --- |
|  | -0.9946 | 0.0489 | 0.000*** |
|  | 0.0040 | 0.0220 | 0.016* |
|  | 1.0025 | -0.0048 | 0.696 |

| NPA x INR  Vol (sensory) | Moderator value | b value | p value |
| --- | --- | --- | --- |
|  | -0.9946 | 0.0480 | 0.000*** |
|  | 0.0040 | 0.0216 | 0.012* |
|  | 1.0025 | -0.0048 | 0.665 |

Note. Outputs of simple slopes analysis for significant interaction effects in primary models. Conditional effects (slopes) of modifiable factors on brain outcome measures are computed for predicted mean ± 1 SD values of the SES moderator (area deprivation index [ADI] or income-to-needs ratio [INR]). PA = physical activity. NPA = non-physical activity. Sleep = sleep duration. CT = cortical thickness. SA = surface area. Vol = volume. rsFC = resting-state functional connectivity. * = p < .05, ** = p < .01, *** = p < .001.

## Figure S7. Association between sleep and brain measures at different levels of income-to-needs ratio (INR).

Panels A to D show associations between sleep and brain structure at different INR levels for: (A) total volume (vol), (B) total surface area (SA), (C) total SA of sensory cortices , (D) total SA of somatosensory/motor cortices, (E) total SA of higher-order association cortices, (F) total vol of sensory cortices, (G) total vol of somatosensory/motor cortices, (H) total vol higher-order association cortices,(I) average cortical thickness (CT) of sensory cortices, (J) average CT of somatosensory/motor cortices. Panels K to L show associations between sleep and resting-state functional connectivity (rsFC) at different levels of INR for: (K) within-higher-order association network rsFC, and (L) between somatosensory/motor network rsFC. For structural outcomes (Panels A-J), the y-axis shows standardized values; for rsFC measures (Panels K-L), the y-axis shows values in their original units *for ease of interpretation*. Slopes represent predicted values at mean ± 1 SD of INR, and shaded areas indicate 95% confidence intervals.

## Figure S8. Association between sleep and resting-state functional connectivity (rsFC) at different levels of area deprivation index (ADI).

(A) average between-network rsFC, and (B) average between somatosensory/motor network rsFC. Slopes represent predicted values at mean ± 1 SD of ADI, and shaded areas indicate 95% confidence intervals. Higher levels of ADI indicate greater disadvantage.

## Figure S9. Association between physical extracurricular activity and brain structure at different levels of income-to-needs ratio (INR).

(A) total volume (vol) of sensory cortices and (B) total surface area (SA) of sensory cortices. The y-axis shows standardized values. Slopes represent predicted values at mean ± 1 SD in INR, and shaded areas indicate 95% confidence intervals.

## Figure S10. Association between non-physical extracurricular activity and total volume (vol) of sensory cortices at different levels of income-to-needs ratio (INR).

The y-axis shows standardized values. Slopes represent predicted values at mean ± 1 SD in INR, and shaded areas indicate 95% confidence intervals.

## Table S10. Interaction effects between socioeconomic status (neighborhood disadvantage and income-to-needs ratio) and modifiable lifestyle factors in models of brain structure and function with site included as a random effect.

| *Predictor* | *Outcome* |  | *b* | *SE* | *df* | *t* | *p* |
| --- | --- | --- | --- | --- | --- | --- | --- |
| *Sleep Duration* | | | | | | | |
| ADI x Sleep | Average between-network rsFC | Global average | -0.018 | 0.008 | 8,215 | -2.26 | 0.024 |
|  |  | Somatosensory/motor | -0.024 | 0.010 | 8,123 | -2.38 | 0.017 |
| INR x Sleep | Surface area | Total | -0.027 | 0.009 | 9,934 | -3.04 | 0.002 |
|  |  | Sensory | -0.030 | 0.009 | 9,917 | -3.29 | 0.001 |
|  |  | Somatosensory/motor | -0.023 | 0.009 | 9,831 | -2.54 | 0.011 |
|  |  | Higher-order association | -0.023 | 0.009 | 9,548 | -2.52 | 0.012 |
|  | Volume | Total | -0.029 | 0.009 | 9,979 | -3.25 | 0.001 |
|  |  | Sensory | -0.033 | 0.009 | 9,941 | -3.65 | 0.000 |
|  |  | Somatosensory/motor | -0.026 | 0.010 | 9,834 | -2.70 | 0.007 |
|  |  | Higher-order association | -0.024 | 0.009 | 9,907 | -2.60 | 0.009 |
|  | Average cortical thickness | Sensory | -0.020 | 0.010 | 9,732 | -2.00 | 0.045 |
|  |  | Somatosensory/motor | -0.017 | 0.010 | 9,680 | -1.69 | 0.091 |
|  | Average within-network rsFC | Higher-order association | -0.028 | 0.010 | 7,973 | -2.91 | 0.003 |
|  | Average between-network rsFC | Somatosensory/motor | 0.025 | 0.011 | 7,863 | 2.27 | 0.023 |
| *Physical Extracurricular activity (Winsorized)* | | | | | | | |
| INR x Physical extracurricular activity | Total surface area | Sensory | -0.023 | 0.009 | 9,844 | -2.66 | 0.008 |
|  | Total volume | Sensory | -0.026 | 0.009 | 9,855 | -2.98 | 0.003 |
| *Non-Physical Extracurricular activity* | | | | | | | |
| INR x Non-physical extracurricular activity | Total volume | Sensory | -0.024 | 0.008 | 10,043 | -2.91 | 0.004 |

Note. The b values represent standardized b coefficients. ADI = area deprivation index. INR = income-to-needs ratio. SE = standard error. df = degrees of freedom. Adj. p = false-discovery rate corrected p value.

Figure S11. Full range association between income-to-needs ratio (INR) and brain measures at different levels of sleep. Panels A to D show associations between INR and brain structure at different sleep levels for: (A) total volume (vol), (B) total surface area (SA), (C) average cortical thickness (CT) of sensory cortices, (D) average CT of somatosensory/motor cortices. Panels E to F show associations between INR and resting-state functional connectivity (rsFC) at different levels of sleep for: (E) within-higher-order association network rsFC, and (F) between somatosensory/motor network rsFC. Volume and surface area (i.e. panel A and B) patterns were identical across the three systems, with system-specific figures available in the supplement (Figure S3). For structural outcomes (Panels A-D), the y-axis shows standardized values; for rsFC measures (Panels E-F), the y-axis shows values in their original units for ease of interpretation. Slopes represent predicted values at mean ± 1 SD of sleep duration, and shaded areas indicate 95% confidence intervals. Due to the high point density, a jitter of 0.1 was added to improve visibility.

Figure S12. Full range association between area deprivation index (ADI) and resting-state functional connectivity (rsFC) at different levels of sleep for: (A) average between-network rsFC, and (B) average between-somatosensory/motor network rsFC. Slopes represent predicted values at mean ± 1 SD of sleep duration, and shaded areas indicate 95% confidence intervals. Higher levels of ADI indicate greater disadvantage. The y-axis shows rsFC values in their original units. Due to the high point density, a jitter of 0.1 was added to improve visibility.

Figure S13. Full range association between income-to-needs ratio (INR) and brain structure at different levels of physical extracurricular activity for: (A) total volume (vol) of sensory cortices and (B) total surface area (SA) of sensory cortices. The y-axis shows standardized values. Slopes represent predicted values at mean ± 1 SD in physical extracurricular activity, and shaded areas indicate 95% confidence intervals. Due to the high point density, a jitter of 0.1 was added to improve visibility.

Figure S14. Full range association between income-to-needs ratio (INR) and total volume (vol) of sensory cortices at different levels of non-physical extracurricular activity. The y-axis shows standardized values. Slopes represent predicted values at mean ± 1 SD in physical extracurricular activity, and shaded areas indicate 95% confidence intervals. Due to the high point density, a jitter of 0.1 was added to improve visibility.

## Table S11. Interaction effects between socioeconomic status (neighborhood disadvantage and income-to-needs ratio) and quadratic sleep duration in models of brain structure and function.

|  |  | ADI x Sleep Duration | | | | | | INR x Sleep Duration | | | | | |
| --- | --- | --- | --- | --- | --- | --- | --- | --- | --- | --- | --- | --- | --- |
| *Outcome* | | *b* | *SE* | *df* | *t* | *p* | *Adj.p* | *b* | *SE* | *df* | *T* | *p* | *Adj.p* |
| Total Surface Area | Total | 0.007 | 0.006299187 | 10,402 | 1.09 | 0.275 | 0.614 | -0.015 | 0.007926709 | 10,076 | -1.92 | 0.055 | 0.356 |
|  | Sensory | 0.005 | 0.006349731 | 10,403 | 0.81 | 0.416 | 0.624 | -0.012 | 0.007994831 | 10,082 | -1.45 | 0.148 | 0.356 |
|  | Somatosensory/motor | 0.007 | 0.006424360 | 10,318 | 1.02 | 0.307 | 0.614 | -0.013 | 0.008145118 | 10,147 | -1.58 | 0.115 | 0.356 |
|  | Higher-order association | 0.008 | 0.006579262 | 10,015 | 1.15 | 0.250 | 0.614 | -0.014 | 0.008306645 | 9,779 | -1.63 | 0.102 | 0.356 |
| Total volume | Total | 0.004 | 0.006336537 | 10,429 | 0.63 | 0.529 | 0.705 | -0.008 | 0.007957982 | 10,025 | -1.00 | 0.315 | 0.472 |
|  | Sensory | 0.001 | 0.006423738 | 10,393 | 0.23 | 0.819 | 0.893 | -0.010 | 0.008084910 | 10,012 | -1.24 | 0.215 | 0.430 |
|  | Somatosensory/motor | -0.001 | 0.006840974 | 10,310 | -0.08 | 0.934 | 0.934 | -0.007 | 0.008722996 | 10,146 | -0.84 | 0.402 | 0.536 |
|  | Higher-order association | 0.002 | 0.006583362 | 10,374 | 0.32 | 0.746 | 0.893 | -0.013 | 0.008342724 | 10,098 | -1.54 | 0.124 | 0.356 |
| Average cortical thickness | Total | -0.011 | 0.006847693 | 10,192 | -1.62 | 0.104 | 0.425 | 0.004 | 0.008736020 | 10,152 | 0.50 | 0.614 | 0.737 |
|  | Sensory | -0.006 | 0.006954230 | 10,224 | -0.85 | 0.395 | 0.624 | -0.001 | 0.008841601 | 10,156 | -0.09 | 0.925 | 0.925 |
|  | Somatosensory/motor | -0.012 | 0.007026048 | 10,151 | -1.74 | 0.081 | 0.425 | 0.009 | 0.008972859 | 10,145 | 1.03 | 0.304 | 0.472 |
|  | Higher-order association | -0.012 | 0.007121038 | 10,189 | -1.62 | 0.106 | 0.425 | 0.001 | 0.009097879 | 10,146 | 0.15 | 0.883 | 0.925 |
| Average within-network rsFC | Total | 0.009 | 0.006846154 | 8,437 | 1.26 | 0.207 | 0.746 | 0.008 | 0.008924309 | 8,406 | 0.89 | 0.376 | 0.626 |
|  | Sensory | 0.006 | 0.007302151 | 8,373 | 0.84 | 0.403 | 0.746 | -0.007 | 0.009535610 | 8,333 | -0.76 | 0.449 | 0.626 |
|  | Somatosensory/motor | 0.005 | 0.007863490 | 8,324 | 0.70 | 0.485 | 0.746 | 0.014 | 0.010206973 | 8,294 | 1.36 | 0.174 | 0.478 |
|  | Higher-order association | 0.008 | 0.006899761 | 8,473 | 1.13 | 0.259 | 0.746 | 0.007 | 0.008995230 | 8,436 | 0.77 | 0.441 | 0.626 |
| Average between-network rsFC | Total | -0.003 | 0.006600657 | 8,436 | -0.46 | 0.644 | 0.746 | -0.004 | 0.008706613 | 8,389 | -0.49 | 0.626 | 0.626 |
|  | Sensory | -0.007 | 0.007915345 | 8,420 | -0.93 | 0.350 | 0.746 | 0.021 | 0.010281479 | 8,362 | 2.02 | 0.043 | 0.159 |
|  | Sensory – somatosensory/ motor | 0.000 | 0.007845246 | 8,410 | 0.02 | 0.988 | 0.988 | 0.022 | 0.010089850 | 8,387 | 2.20 | 0.028 | 0.155 |
|  | Sensory – higher-order association | -0.003 | 0.007401102 | 8,395 | -0.41 | 0.678 | 0.746 | -0.006 | 0.009637826 | 8,379 | -0.67 | 0.502 | 0.626 |
|  | Somatosensory/ motor | -0.006 | 0.007940383 | 8,349 | -0.77 | 0.440 | 0.746 | 0.006 | 0.010242457 | 8,344 | 0.56 | 0.578 | 0.626 |
|  | Somatosensory/ motor – higher-order association | 0.004 | 0.007311698 | 8,323 | 0.53 | 0.599 | 0.746 | -0.025 | 0.009510788 | 8,317 | -2.67 | 0.008 | 0.083 |
|  | Higher-order association | -0.004 | 0.007037508 | 8,403 | -0.56 | 0.573 | 0.746 | 0.005 | 0.009231265 | 8,353 | 0.50 | 0.615 | 0.626 |

Note. The b values represent standardized b coefficients. ADI = area deprivation index. INR = income-to-needs ratio. SE = standard error. df = degrees of freedom. Adj. p = false-discovery rate corrected p value.

## Table S12. Joint distributions of SES and lifestyle factors.

| **Sleep Duration (hours/night)** | | | | | | |  | |  |
| --- | --- | --- | --- | --- | --- | --- | --- | --- | --- |
| **ADI Tertile** | <5 | 5-7 | 7-8 | 8-9 | 9-11 | Total | | | |
| Low disadvantage | 0 (0%) | 36 (1.0%) | 229 (6.3%) | 1,216 (33%) | 2,163 (59%) | 3,644 (100%) | | | |
| Medium disadvantage | 6 (0.2%) | 76 (2.1%) | 382 (11%) | 1,318 (36%) | 1,856 (51%) | 3,638 (100%) | | | |
| High disadvantage | 26 (0.7%) | 200 (5.5%) | 691 (19%) | 1,531 (42%) | 1,176 (32%) | 3,624 (100%) | | | |
| Total | 32 (0.3%) | 312 (2.9%) | 1,302 (12%) | 4,065 (37%) | 5,195 (48%) | 10,906 (100%) | | | |
| **INR Tertile** |  |  |  |  |  |  | | | |
| Low INR | 23 (0.7%) | 207 (5.9%) | 677 (19%) | 1,502 (43%) | 1,107 (31%) | 3,516 (100%) | | | |
| Medium INR | 3 (<0.1%) | 70 (2.0%) | 341 (9.6%) | 1,283 (36%) | 1,842 (52%) | 3,539 (100%) | | | |
| High INR | 1 (<0.1%) | 28 (0.8%) | 193 (5.5%) | 1,064 (30%) | 2,248 (64%) | 3,534 (100%) | | | |
| Total | 27 (0.3%) | 305 (2.9%) | 1,211 (11%) | 3,849 (36%) | 5,197 (49%) | 10,589 (100%) | | | |
| **Extracurricular Physical Activity Quintile** | | | | | | | |  |  |
| **ADI Tertile** | 1 | 2 | 3 | 4 | 5 | Total | | | |
| Low disadvantage | 354 (9.7%) | 647 (18%) | 814 (22%) | 900 (25%) | 928 (25%) | 3,643 (100%) | | | |
| Medium disadvantage | 664 (18%) | 780 (21%) | 753 (21%) | 730 (20%) | 710 (20%) | 3,637 (100%) | | | |
| High disadvantage | 1,163 (32%) | 754 (21%) | 614 (17%) | 551 (15%) | 542 (15%) | 3,624 (100%) | | | |
| Total | 2,181 (20%) | 2,181 (20%) | 2,181 (20%) | 2,181 (20%) | 2,180 (20%) | 10,904 (100%) | | | |
| **INR Tertile** |  |  |  |  |  |  | | | |
| Low INR | 1,290 (37%) | 752 (21%) | 595 (17%) | 468 (13%) | 410 (12%) | 3,515 (100%) | | | |
| Medium INR | 566 (16%) | 767 (22%) | 761 (22%) | 722 (20%) | 723 (20%) | 3,539 (100%) | | | |
| High INR | 262 (7.4%) | 599 (17%) | 761 (22%) | 927 (26%) | 984 (28%) | 3,533 (100%) | | | |
| Total | 2,118 (20%) | 2,118 (20%) | 2,117 (20%) | 2,117 (20%) | 2,117 (20%) | 10,587 (100%) | | | |
| **Extracurricular Non-Physical Activity Quintile** | | | | | | | |  |  |
| **ADI Tertile** | 1 | 2 | 3 | 4 | 5 | Total | | | |
| Low disadvantage | 559 (15%) | 493 (14%) | 745 (20%) | 937 (26%) | 909 (25%) | 3,643 (100%) | | | |
| Medium disadvantage | 703 (19%) | 738 (20%) | 744 (20%) | 749 (21%) | 703 (19%) | 3,637 (100%) | | | |
| High disadvantage | 919 (25%) | 950 (26%) | 692 (19%) | 495 (14%) | 568 (16%) | 3,624 (100%) | | | |
| Total | 2,181 (20%) | 2,181 (20%) | 2,181 (20%) | 2,181 (20%) | 2,180 (20%) | 10,904 (100%) | | | |
| **INR Tertile** |  |  |  |  |  |  | | | |
| Low INR | 997 (28%) | 961 (27%) | 602 (17%) | 454 (13%) | 501 (14%) | 3,515 (100%) | | | |
| Medium INR | 731 (21%) | 618 (17%) | 720 (20%) | 720 (20%) | 750 (21%) | 3,539 (100%) | | | |
| High INR | 390 (11%) | 539 (15%) | 795 (23%) | 943 (27%) | 866 (25%) | 3,533 (100%) | | | |
| Total | 2,118 (20%) | 2,118 (20%) | 2,117 (20%) | 2,117 (20%) | 2,117 (20%) | 10,587 (100%) | | | |

Note: Sample sizes vary across joint distributions because participants were excluded only if they were missing the SES, moderator, or covariate variable relevant to that distribution. Participants were required to have an MRI model but not sMRI- or fMRI-specific variables, as these outcomes were analyzed separately. ADI = area deprivation index. INR = income-to-needs ratio.
